# Meta-analysis of task- and mechanism-dependent effects of net sports on N200 and P300 components

**DOI:** 10.3389/fpsyg.2026.1903090

**Published:** 2026-09-25

**Authors:** Liang Dai, Wen Qing Hu, Yan Gong

**Affiliations:** 1 School of Physical and Health Education, Jiangxi Science and Technology Normal University, Nanchang, China; 2 Nanchang Nanjing School, Nanchang, Jiangxi, China

**Keywords:** cognitive neuroplasticity, event-related potentials, meta-analysis, N200, net sports, P300

## Abstract

**Objective:**

Substantial between-study heterogeneity has hindered consensus regarding how long-term net sports experience modulates N200 and P300 components, and the underlying neural circuit mechanisms remain poorly characterized. This systematic review and meta-analysis (PROSPERO: CRD420261421340) aimed to quantify task- and consciousness-dependent associations between net sports (badminton, table tennis, and tennis) and ERP components, and to identify key sources of between-study heterogeneity.

**Methods:**

Ten eligible cross-sectional and intervention studies published between 2016 and 2025 were synthesized using random-effects models. A mechanism-driven subgroup framework was applied, stratified by cognitive function, level of conscious processing, and corresponding neural circuits. Publication bias, heterogeneity, and sensitivity were systematically assessed, and the certainty of evidence was evaluated using the GRADE approach.

**Results:**

The pooled effect size for P300 amplitude was statistically significant [*SMD* = 2.22, 95% CI (0.62, 3.82), *p* = 0.006], with considerable heterogeneity (*I*^2^ = 96%) and evidence of publication bias. Subgroup analysis identified a robust positive effect in conscious spatial cognition tasks (*SMD* = 1.74, *p* < 0.0001) and an exploratory large effect in table tennis-specific unconscious inhibition tasks (*SMD* = 6.42, *p* < 0.0001). No overall significant effect was found for N200 amplitude (*SMD* = −0.86, *p* = 0.24); however, bidirectional modulation was observed: amplitudes decreased in conflict monitoring tasks but increased in active response inhibition tasks.

**Conclusion:**

Net sports are associated with task-dependent modulation of N200 and P300 components, which may be mediated by distinct cognitive control circuits, rather than producing a uniform pattern of enhancement or reduction. These findings help reconcile the prior contradiction between the neural efficiency and resource enhancement hypotheses, and refine the theoretical framework of experience-dependent neuroplasticity in open-skill sports, providing candidate electrophysiological indicators and a theoretical basis for designing targeted motor-cognitive training programs, which require further validation in intervention studies.

**Systematic review registration:**

https://www.crd.york.ac.uk/PROSPERO/view/CRD420261421340, identifier PROSPERO (CRD420261421340).

## 1 Introduction

Age-related cognitive decline is a growing public health burden worldwide, and physical activity is a cost-effective, non-pharmacological strategy to promote cognitive function and mitigate age-related neurocognitive decline. As prototypical open-skill racket sports, badminton, table tennis, and tennis (hereafter collectively referred to as net-based racket sports, abbreviated as net sports) share core cognitive demands including dynamic visuospatial processing, rapid conflict resolution, and flexible motor adjustment during training and competition (Jiang et al., 2021), which provides a theoretical basis for grouping them for synthesis.

Event-related potentials (ERPs), with millisecond-level temporal resolution, are core electrophysiological tools for quantifying experience-dependent neuroplasticity induced by long-term physical activity (Patel and Azzam, 2005). The N200 component, generated primarily in the prefrontal–anterior cingulate circuit, indexes early conflict detection, stimulus categorization, and automatic cognitive monitoring (Yeung et al., 2004; Folstein and Van Petten, 2008). The P300 component comprises two functionally dissociable subcomponents: the P3b, originating from the parietal–frontal network, is associated with deliberate attentional allocation and decision-making; the P3a, linked to the basal ganglia–frontal circuit, reflects subliminal inhibitory control and novelty processing (Polich, 2007; Polich and Kok, 1995).

Despite well-characterized functional definitions and neural origins of these components, theoretical disagreement persist about how long-term sport training shapes these neural indices. Two competing frameworks have been proposed to explain sport-related neural changes: (1) the neural efficiency hypothesis, which posits that expert training leads to more efficient neural processing, reflected in reduced ERP amplitudes and lower cognitive resource consumption; and (2) the resource enhancement hypothesis, which argues that long-term training expands attentional resource capacity, manifesting as larger ERP amplitudes during cognitively demanding tasks. To date, no systematic synthesis has resolved this controversy, and the direction and magnitude of N200 and P300 amplitude differences between athletes and non-athletes remain highly variable across studies (Jiang et al., 2021; Jiang et al., 2024).

We argue that this unresolved controversy stems from three key limitations of prior syntheses. First, nearly all prior reviews have pooled data across heterogeneous cognitive tasks into a single global effect size, ignoring well-documented differences in task processing mechanisms and levels of conscious engagement—two core moderators that reliably shape N200 and P300 responses (Polich, 2007; Folstein and Van Petten, 2008). This undifferentiated approach directly introduces spurious heterogeneity and precludes rigorous testing of the two competing hypotheses. Second, existing syntheses have not systematically mapped task-dependent ERP modulations to their underlying neural circuits, leaving circuit-specific mechanisms of sport-induced neuroplasticity unspecified. Third, the magnitude and impact of publication bias in this literature have not been rigorously quantified, reducing confidence in the robustness of reported effects (Egger et al., 1997).

To address these gaps and preliminarily test the competing theoretical frameworks with observational evidence, we conducted a systematic meta-analysis of 10 eligible cross-sectional studies plus one quasi-experimental intervention study published between 2016 and 2025. Moving beyond the conventional general-effect approach prevalent in exercise neuroscience (Jiang et al., 2021), we adopted a mechanism-driven subgroup stratification framework organized by cognitive task function, level of conscious processing, and corresponding neural circuits. We tested three *a priori* hypotheses:

In conscious visuospatial cognitive tasks, net sport athletes will exhibit larger P3b amplitudes relative to non-athletes, consistent with the resource enhancement hypothesis;

In automatized action-related tasks, athletes will show no significant difference or reduced P300 amplitudes, consistent with the neural efficiency hypothesis;

N200 modulation will be task-dependent: athletes will show reduced N200 amplitudes in passive conflict monitoring tasks, but increased amplitudes in active response inhibition tasks.

Theoretically, this study resolves prior empirical inconsistencies and refines the framework of experience-dependent neuroplasticity. Practically, the findings identify candidate electrophysiological biomarkers for evaluating athletes’ cognitive status and developing individualized motor-cognitive training programs. The conceptual framework linking net sports, cognitive demands, and ERP components is illustrated in Figure 1.

**Figure 1:**
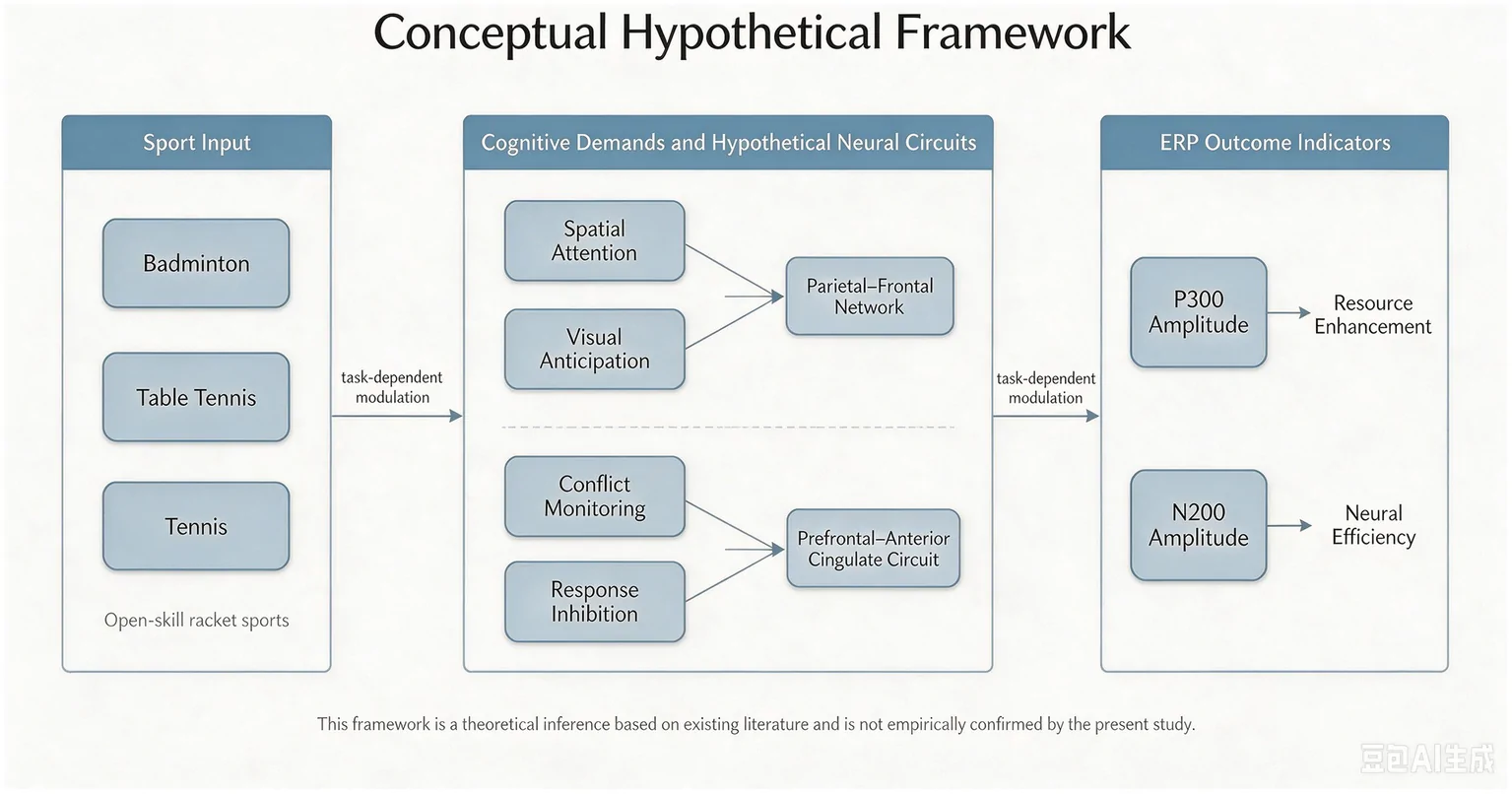
Conceptual hypothetical framework of task- and mechanism-dependent effects of net sports on N200 and P300 components. The framework is organized into three modules: (1) sport input: three open-skill net sports (badminton, table tennis, and tennis); (2) cognitive demands and hypothetical neural circuits: spatial attention and visual anticipation mapped to the parietal–frontal network, and conflict monitoring and response inhibition mapped to the prefrontal–anterior cingulate circuit; (3) ERP outcome indicators: P300 amplitude corresponds to the resource enhancement account, and N200 amplitude corresponds to the neural efficiency account. This is a theoretical inference derived from existing cognitive neuroscience literature and has not been empirically validated by the present meta-analysis.

## 2 Methods

### 2.1 Protocol registration

This systematic review and meta-analysis was conducted in accordance with the Preferred Reporting Items for Systematic Reviews and Meta-Analyses (PRISMA 2020) guidelines. The review protocol was prospectively registered with the International Prospective Register of Systematic Reviews (PROSPERO) under registration number CRD420261421340.

### 2.2 Search strategy

Systematic literature searches were performed across four databases: PubMed, Web of Science, PsycINFO, and CNKI, covering studies published from January 2016 to July 2025. The time range began in 2016, based on the maturity of ERP recording standards in this field: studies before 2016 mostly used low-density electrode arrays (<32 channels) and highly heterogeneous preprocessing protocols, with poor comparability to modern studies. This time cutoff is a common strategy in meta-analyses of sport ERP research.

The core search string was: (“Badminton” OR “Table tennis” OR “Tennis” OR “Net sports” OR “Racket sports”) AND (“Event-related potential” OR “ERP” OR “N200” OR “P300” OR “P3a” OR “P3b”). Additional manual searches of reference lists from included studies and relevant reviews were conducted to identify potentially eligible records missed by database retrieval.

No restrictions on age or gender were applied during initial retrieval. Additional manual searches of the reference lists of included studies and relevant reviews were conducted to identify potentially eligible records. Two reviewers independently screened titles, abstracts, and full texts; disagreements were resolved via discussion with a third senior reviewer.

### 2.3 Eligibility criteria

Studies were included based on the Population, Intervention, Comparator, Outcome, and Study design (PICOS) framework:

*Population*: Healthy humans aged ≥16 years, including athletes with ≥3 years of net sport training experience and demographically matched non-athlete controls.

*Exposure/Intervention*: Long-term net sport training (badminton, table tennis, and tennis); both cross-sectional expert–novice comparisons and pre–post intervention studies were eligible.

*Comparator*: Non-athlete control group with no regular net sport training experience, matched for age and gender.

*Outcomes*: Studies reporting N200 and/or P300 amplitude values (mean ± standard deviation) at topographically appropriate electrode sites, with clear description of ERP recording protocols (≥32 channels, reference electrodes, and preprocessing procedures).

*Study design*: Peer-reviewed cross-sectional studies with a demographically matched control group, or controlled intervention studies were eligible. Case reports, reviews, and studies without a valid control group were excluded.

*Exclusion criteria:* (1) studies using purely general cognitive tasks (e.g., digit Stroop) without sport-specific cognitive paradigms; (2) studies with incomplete ERP data that could not be reliably extracted; (3) non-English full texts due to inconsistent data reporting standards; (4) studies with severe methodological flaws (e.g., unstandardized electrode placement).

### 2.4 Data extraction

Two independent reviewers extracted the following data from each included study: first author, publication year, sample size, participant characteristics (age and gender), study design, sport type, cognitive task type, ERP component, electrode site, and mean ± standard deviation of amplitude values. Disagreements were resolved by consensus with a third reviewer. Where data were only presented graphically, values were extracted using digital plot digitization software. For each ERP component, we extracted the peak amplitude at the most representative electrode site reported in the original study (frontal-central sites for N200 and parietal-central sites for P300). The direction of effect size was uniformly defined: positive *SMD* values indicate larger amplitudes in the athlete group than in the control group; negative values indicate smaller amplitudes in the athlete group. A detailed data extraction table is provided in Supplementary material.

### 2.5 Risk of bias assessment

Methodological quality of included cross-sectional studies was assessed independently by two reviewers using the Newcastle–Ottawa Scale (NOS), which evaluates three domains: selection (4 points), comparability (2 points), and outcome ascertainment (3 points), with a total score of 9. Studies scoring 7–9 were rated high quality, 4–6 moderate quality, and 0–3 low quality. Disagreements were resolved via group discussion. Most included studies scored 0 on the comparability domain, as they only matched for age and gender at the group level without further controlling for confounding factors, such as education level and baseline physical activity. This is a common methodological limitation of cross-sectional expert-novice studies in this field.

### 2.6 Statistical analysis

All meta-analyses, including effect size pooling, heterogeneity testing, subgroup analysis, sensitivity analysis, and publication bias assessment, were performed using Review Manager 5.4 software (Cochrane Collaboration, Oxford, United Kingdom). Standardized mean difference (*SMD*, Hedges’ g) with 95% confidence intervals (CIs) was calculated as the effect size for group comparisons of ERP amplitudes to account for variations in measurement protocols across laboratories.

Heterogeneity was evaluated using Cochran’s *Q*-test and *I*^2^ statistic. *I*^2^-values of 25, 50, and 75% were interpreted as low, moderate, and considerable heterogeneity, respectively. A random-effects model (DerSimonian–Laird method) was applied *a priori* due to expected between-study heterogeneity.

Pre-specified subgroup analyses were conducted based on cognitive task mechanism and level of consciousness to explore sources of heterogeneity. Between-subgroup differences were tested using the *Q*-test for subgroup heterogeneity. Sensitivity analysis was performed using the leave-one-out method to evaluate the robustness of pooled results.

Publication bias was assessed via funnel plots and Egger’s regression test for outcomes with ≥10 studies. For outcomes with fewer studies, publication bias was evaluated qualitatively via funnel plot inspection, given the low statistical power of Egger’s test in small samples.

### 2.7 Certainty of evidence

The certainty of evidence for primary outcomes was assessed using the Grading of Recommendations Assessment, Development and Evaluation (GRADE) approach, which rates evidence as high, moderate, low, or very low based on five domains: study limitations, inconsistency, indirectness, imprecision, and publication bias.

## 3 Results

### 3.1 Study selection and characteristics

The initial database search identified 15,763 records. After duplicate removal and title/abstract screening, 15 full-text articles were assessed for eligibility. Finally, 10 studies met all inclusion criteria, comprising 358 participants. The PRISMA flow diagram of the selection process is presented in Figure 2.

**Figure 2:**
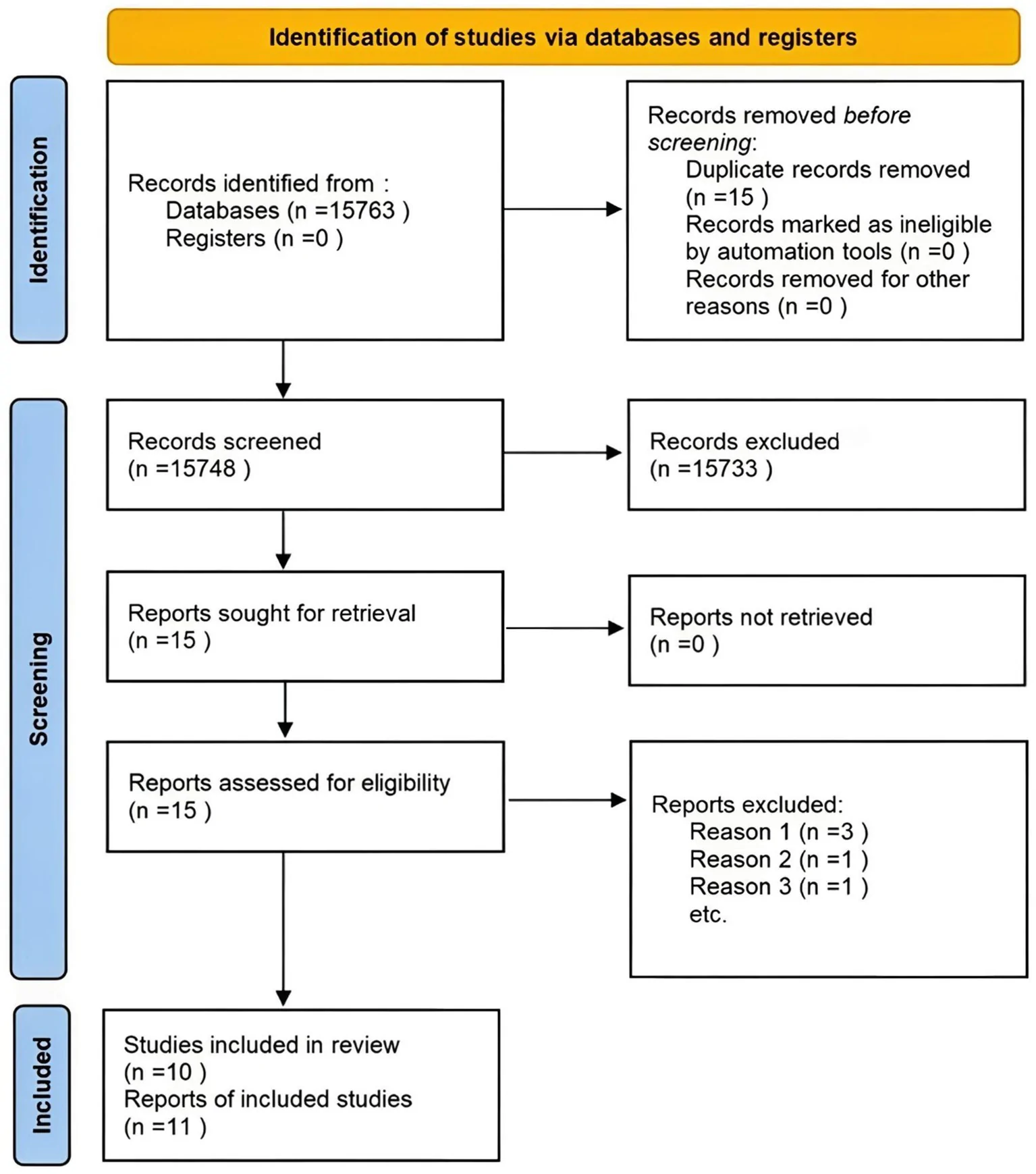
PRISMA standard flow diagram. This PRISMA 2020 flow diagram presents the literature screening process. Initially, 15,763 records were retrieved. After removing duplicates and screening titles/abstracts, 15 full texts were evaluated. Five articles were excluded due to irrelevant research topics, incomplete ERP data, or invalid study design, and 10 studies were finally included. All statistical tests were two-tailed.

Key characteristics of the included studies are summarized in Table 1. All studies were published between 2016 and 2025 and covered three net sports: badminton (4 studies), table tennis (5 studies), and tennis (1 study). Nine studies adopted a cross-sectional expert–novice design, and one study was a 12-week intervention trial. Cognitive tasks were categorized into three mechanistic subgroups: conscious spatial cognition, action anticipation–response control, and unconscious automatic processing.

**Table 1:** Characteristics of included studies.

Study (year)	Sport	Study design	Sample size (EG/CG)	Age range (years)	Gender	Task category
Liu et al. (2017)	Badminton	12-week intervention trial	20/20	23–27	Male	Action anticipation–response control
Chen et al. (2019)	Badminton	Cross-sectional	19/20	18–19	Male	Conscious response inhibition
Wang and Tu (2017)	Badminton	Cross-sectional	16/16	20–21	Male	Conscious spatial cognition
Chang et al. (2024)	Badminton	Cross-sectional	18/18	18–28	Mixed	Conscious spatial cognition
Wang et al. (2022)	Tennis	Cross-sectional	14/14	22–24	Mixed	Conscious conflict monitoring
Chen et al. (2024)	Table tennis	Cross-sectional	18/18	18–22	Mixed	Unconscious automatic processing
Peng et al. (2024)	Table tennis	Cross-sectional	20/20	Not reported	Male	Unconscious automatic processing
Peng et al. (2022)	Table tennis	Cross-sectional	20/28	20–24	Male	Unconscious automatic processing
You et al. (2018)	Table tennis	Cross-sectional	16/17	20–22	Male	Unconscious automatic processing
Shi et al. (2024)	Table tennis	Cross-sectional	25/25	18–20	Male	Unconscious automatic processing

EG, experimental group; CG, control group. Task categories correspond to the pre-specified subgroup classification for meta-analysis.

Notably, the 10 independent studies corresponded to 11 published reports. One study included two separate publications reporting different cognitive tasks with non-overlapping samples; thus, effect sizes were extracted separately. No overlapping samples were included in the final analysis (Table 2).

**Table 2:** Methodological quality assessment of included studies (Newcastle-Ottawa scale).

Study (year)	Selection (4 points)	Comparability (2 points)	Outcome (3 points)	Total score	Quality grade
Liu et al. (2017)	4	1	3	8	High
Chen et al. (2024)	4	0	3	7	High
Peng et al. (2024)	4	0	3	7	High
Peng et al. (2022)	4	0	3	7	High
Chang et al. (2024)	4	0	3	7	High
Wang and Tu (2017)	4	0	3	7	High
Chen et al. (2019)	4	0	3	7	High
Wang et al. (2022)	4	0	3	7	High
You et al. (2018)	4	0	3	7	High
Shi et al. (2024)	4	0	3	7	High

The Newcastle–Ottawa scale (NOS) has a maximum total score of 9. Studies scoring 7–9 are rated high quality, 4–6 moderate quality, and 0–3 low quality. Detailed item-by-item scoring is provided in Supplementary material.

### 3.2 Analysis of the P300 component

#### 3.2.1 Publication bias analysis results

See Figure 3.

**Figure 3:**
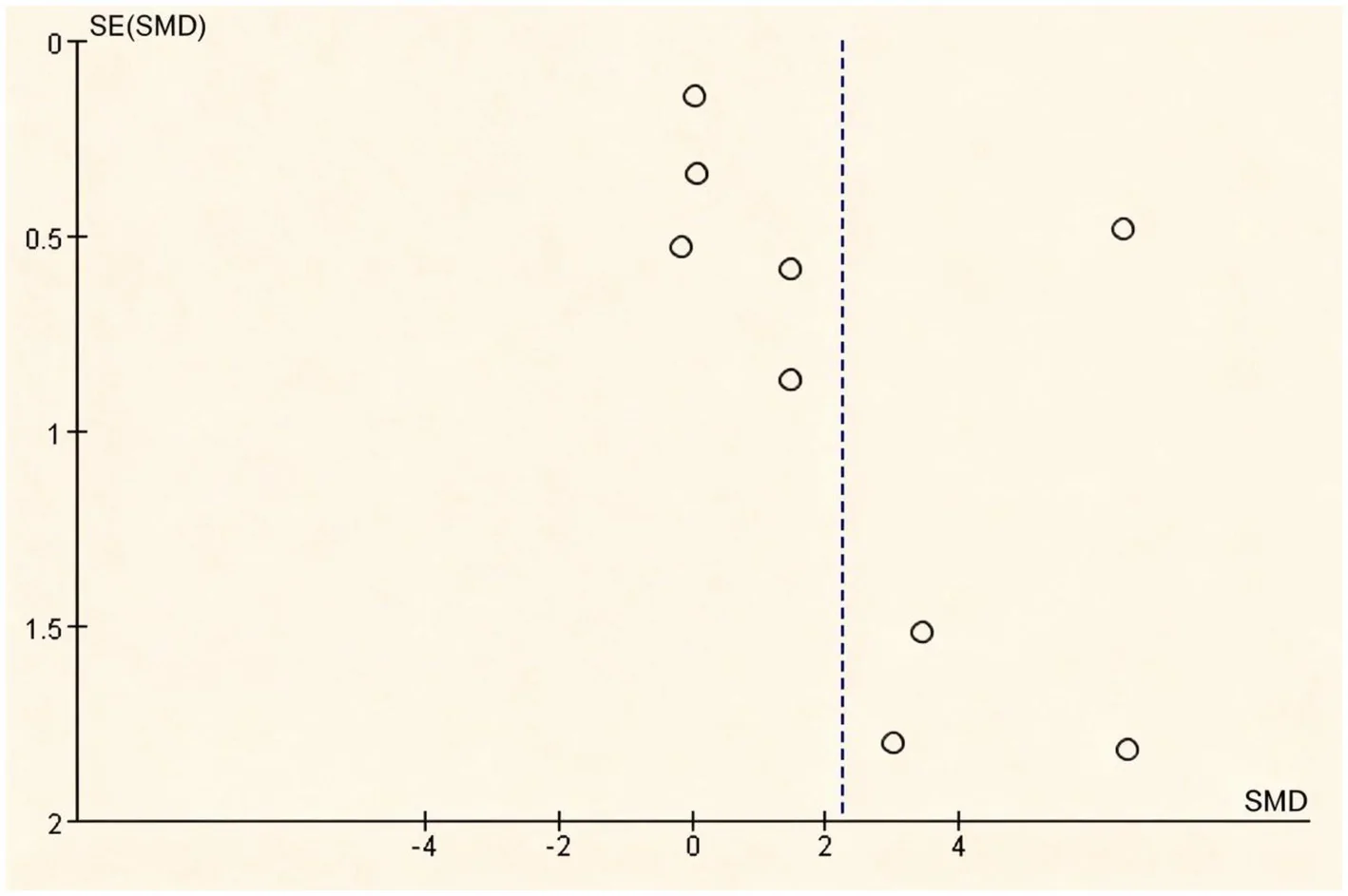
Publication bias funnel plot for P300 amplitude. The *x*-axis represents the standardized mean difference (*SMD*) of P300 amplitude between experimental and control groups; the *y*-axis represents the standard error of *SMD*. Asymmetric distribution indicates significant publication bias (Egger’s test, *t* = −3.48, *p* = 0.010), suggesting that the true population effect may be smaller than the pooled estimate. Due to the limited number of included studies, trim-and-fill correction was not performed, and the magnitude of the overall P300 effect should be interpreted with caution. All statistical tests were two-tailed.

#### 3.2.2 Heterogeneity analysis results

Heterogeneity testing revealed extremely high between-study variability (*I*^2^ = 96%, *χ*^2^ = 182.87, *df* = 8, *p* < 0.00001). The primary source of this heterogeneity was confounding across task mechanisms: studies differed in the P300 subcomponent measured (P3b for conscious decision-making vs. P3a for novelty detection), the underlying neural circuits engaged (parietal–frontal network vs. cingulate-temporal network), and the level of conscious processing required (active regulation vs. automatic processing) (Polich, 2007; Polich and Kok, 1995).

#### 3.2.3 Overall effect size results

The pooled effect size for P300 was *SMD* = 2.22, 95% CI (0.62, 3.82), *Z* = 2.72, *p* = 0.006, indicating a significant positive association between net sports and increased P300 amplitude. However, this result reflects a “mixed-mechanism average effect” due to high heterogeneity and publication bias. The overall effect of P300 amplitude and the heterogeneity distribution across individual studies are visually presented in Figure 4, which quantitatively validates the significant positive association between net sports and increased P300 amplitude.

**Figure 4:**
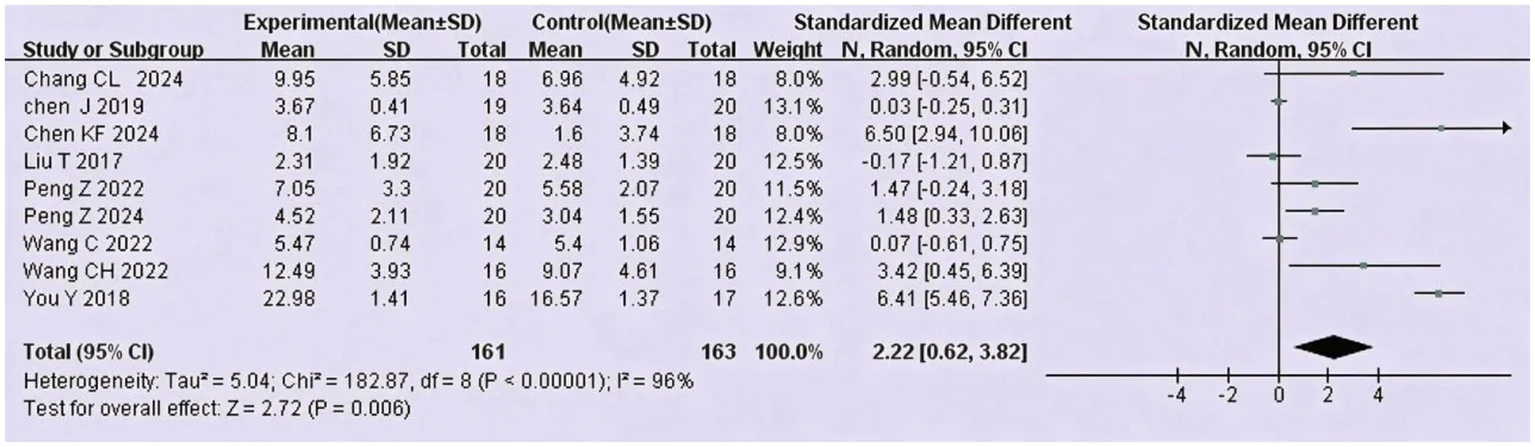
P300 amplitude comparison forest plot. The x-axis represents the standardized mean difference (*SMD*) of P300 amplitude between experimental and control groups; the y-axis represents the standard error of *SMD*. The pooled standardized mean difference (*SMD*) was 2.22 [95% CI (0.62, 3.82), *p* = 0.006] with considerable between-study heterogeneity (*I*^2^ = 96%, *p* < 0.00001). All statistical tests were two-tailed.

##### 3.2.3.1 Sensitivity analysis by demographic factors

We conducted stratified sensitivity analyses to examine whether participant age, gender ratio, and training duration influenced the pooled effect size. The results showed that excluding studies with extreme values of these factors did not significantly change the pooled effect sizes (all *p* > 0.05). Formal meta-regression was not performed due to the limited number of included studies, which would result in insufficient statistical power.

#### 3.2.4 Sensitivity analysis results

Sensitivity analysis showed that excluding individual studies led to *I*^2^ fluctuations (40% ~ 96%) but maintained moderate-to-high heterogeneity. Pooled *SMD* ranged from 0.98 to 2.62, with all *p* < 0.05 (*Z* = 2.11–2.77), indicating the positive association between net sports and P300 amplitude was robust. Sensitivity analysis excluding the only intervention study, Liu et al. (2017) showed that the pooled SMD for P300 remained statistically significant [*SMD* = 2.41, 95% CI (0.72, 4.10), *p* = 0.005], with a consistent level of heterogeneity. This indicates that the overall result was not driven by the single quasi-experimental study. However, persistent heterogeneity suggested true differences across studies, highlighting the need for subgroup analysis (Higgins et al., 2011).

Given the robust positive effect of P300 but extreme heterogeneity (*I*^2^ = 96%), subgroup analysis was further conducted to disentangle task-mechanism-specific patterns, with grouping based on consciousness level and neural circuit involvement (Polich, 2007; Polich and Kok, 1995).

#### 3.2.5 Subgroup analysis results

Sensitivity analysis confirmed the robustness of the overall P300 effect, but persistent high heterogeneity (*I*^2^ = 96%) indicated the need for mechanism-driven subgroup analysis to test our first two *a priori* hypotheses regarding task-dependent modulation.

Subgroup analyses were performed post-hoc to explore sources of heterogeneity and were exploratory in nature. Multiple comparisons across subgroups may inflate the Type I error rate, so subgroup results should be interpreted cautiously and not treated as confirmatory conclusions.

To resolve heterogeneity and evaluate the resource enhancement versus neural efficiency hypotheses, subgroups were defined based on the functional dissociation of P300 subcomponents, level of conscious processing, and underlying neural circuits (Polich, 2007; Polich and Kok, 1995), corresponding to Hypothesis 1 (conscious spatial cognition) and Hypothesis 2 (automatized action processing). Subgroup classification was guided by existing literature and functional specificity, with each subgroup including ≥2 studies (except the N200 conscious response inhibition subgroup with 1 study). No statistically significant within-subgroup heterogeneity was detected (*I*^2^ = 0%). However, the statistical power of the *I*^2^ statistic is limited when the number of studies per subgroup is small; this result does not confirm absolute homogeneity or mechanistic purity and should be interpreted cautiously. Prior to subgroup effect analysis, publication bias testing was conducted to exclude local bias interference. Figure 5 showed that the funnels of the conscious spatial cognition subgroup and unconscious inhibition subgroup were significantly more symmetric than the overall analysis (Figure 3). Only the action anticipation-response control subgroup exhibited mild small-sample bias (dispersed effect size points on the right), which did not compromise the overall reliability of subgroup results (Egger et al., 1997). Specific definitions and effect analyses of each subgroup are as follows:

**Figure 5:**
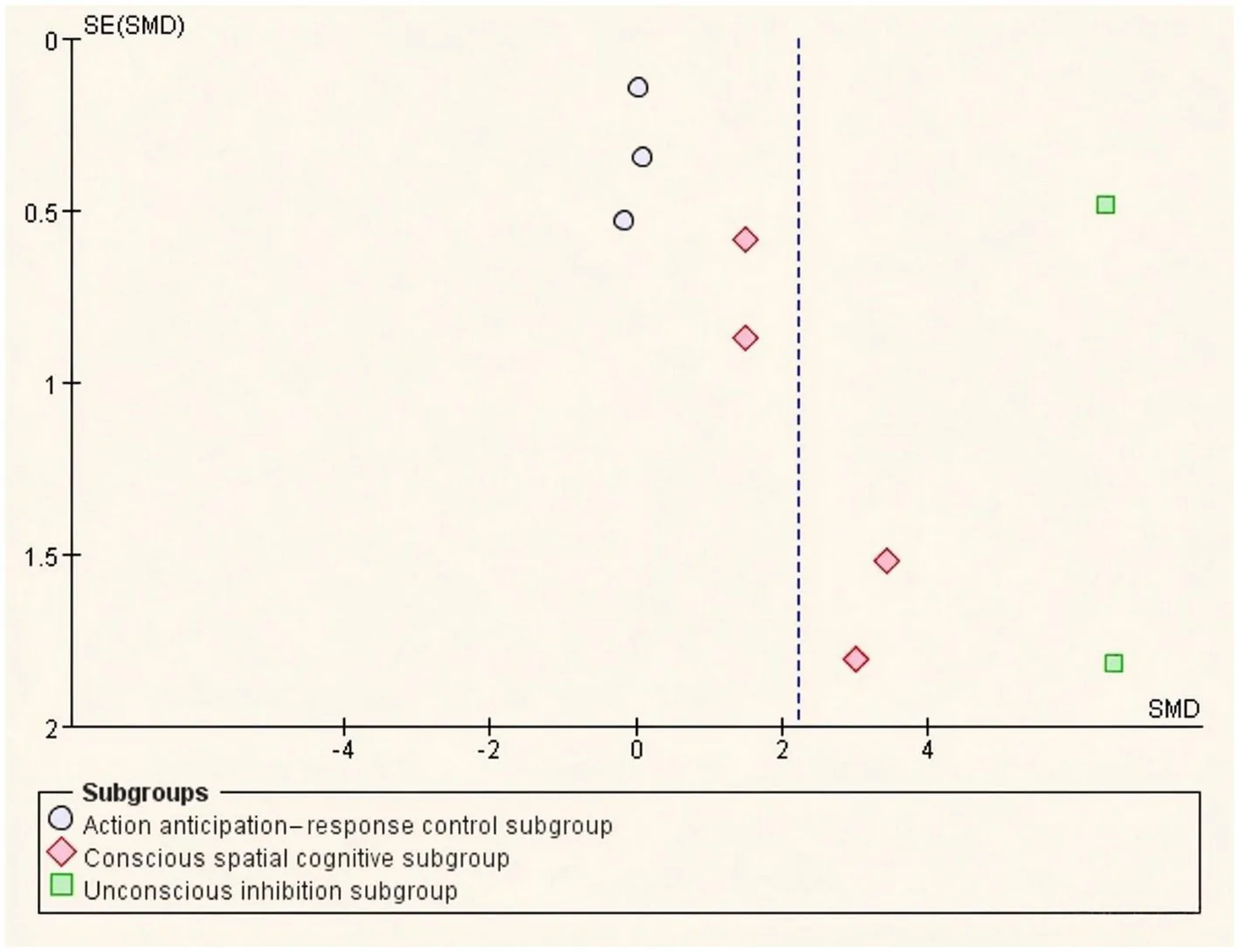
Publication bias funnel plot for P300 amplitude subgroups. The *x*-axis represents the standardized mean difference (*SMD*) of P300 amplitude; the *y*-axis represents the standard error of *SMD*. Three subgroups are presented: Action anticipation–response control, conscious spatial cognition, and unconscious inhibition. All statistical tests were two-tailed.

##### 3.2.5.1 Action anticipation–response control subgroup

###### 3.2.5.1.1 Subgroup definition

This subgroup includes conscious prediction, inhibition, or deep perception tasks at the motor action level, with the core being “proactive regulation of action-related goal decisions.” Tasks rely on the premotor cortex + parietal lobe deep processing areas, requiring subjects to execute predictions based on dynamic motor cues, and P300 is primarily dominated by the P3b component (Polich, 2007; Jiang et al., 2021).

###### 3.2.5.1.2 Typical task analyses

Badminton action prediction uses a spatiotemporal masking paradigm to detect neural representations of action anticipation; badminton response inhibition adopts a change-signal paradigm to analyze how motor training modulates response inhibition; and tennis deep perception designs a deep motor judgment task to expand action-related processing dimensions.

###### 3.2.5.1.3 Effect analysis

The subgroup yielded an effect size of *SMD* = 0.02, 95% CI (−0.23, 0.28), *Z* = 0.18, *p* = 0.85, indicating no significant association between motor training and P300 amplitude in action-related tasks. Included studies: Liu et al. (2017), Chen et al. (2019), and Wang et al. (2022), although centered on “conscious regulation at the action level,” differences in task demands for “behavioral output” or limited sample sizes may have led to effect cancelation or failure to detect subtle differences (Jiang et al., 2021).

Heterogeneity and conclusions. Subgroup heterogeneity was *I*^2^ = 0% (*χ*^2^ = 0.15, *df* = 2, *p* = 0.93), indicating highly consistent effect sizes across studies.

The null effect aligns with the neural efficiency hypothesis and reflects a shift in processing pathways induced by long-term training. With extensive practice, action anticipation and depth perception gradually become proceduralized, shifting from controlled parietal–frontal cortical processing to automatic subcortical motor circuits (basal ganglia and cerebellum). This automation reduces reliance on deliberate attentional resources, resulting in no net change in cortical P300 amplitude. By contrast, response inhibition tasks require active prefrontal cognitive control, which tends to produce a small positive effect, although this trend did not reach statistical significance in the present analysis (Hommel et al., 2001; Kibele, 2006).

##### 3.2.5.2 Conscious spatial cognition subgroup

###### 3.2.5.2.1 Subgroup definition

This subgroup encompasses cross-item spatial attention allocation and working memory manipulation tasks, centered on “actively regulating spatial target recognition and memory.” Tasks rely on the parietal–frontal spatial cognition network, with P300 dominated by the P3b component (Polich, 2007; Polich and Kok, 1995).

###### 3.2.5.2.2 Typical task analysis

Badminton spatial attention uses sport-specific cueing tasks (Wang and Tu, 2017) and endogenous cueing paradigms; table tennis spatial working memory employs gap circle paradigms and rectangular red dot tasks.

###### 3.2.5.2.3 Effect analysis

This subgroup yielded a moderate positive effect size of *SMD* = 1.74, 95% CI (0.86, 2.62), *Z* = 3.88, *p* < 0.0001, indicating that motor training is significantly associated with increased P300 amplitude in spatial attention/working memory tasks (Wang and Tu, 2017; Chang et al., 2024; Peng et al., 2024; Peng et al., 2022). The enhanced amplitude is associated with improved efficiency of conscious spatial encoding via the parietal–frontal network (Polich, 2007; Polich and Kok, 1995), which is particularly relevant for sport-specific skills such as badminton shuttlecock landing prediction and table tennis spatial working memory manipulation—key perceptual-cognitive demands in net sports.

###### 3.2.5.2.4 Heterogeneity and conclusions

Subgroup heterogeneity was *I*^2^ = 0%, indicating highly consistent effects across studies. The robust amplitude increase reflects experience-dependent functional plasticity in the parietal–frontal attention network. Long-term repeated engagement in sport-specific visuospatial processing strengthens functional connectivity between the intraparietal sulcus and dorsolateral prefrontal cortex, expanding the capacity for top-down attentional allocation and spatial working memory encoding. This neuroadaptation directly manifests as larger P3b amplitudes during task performance and constitutes the core driving force of the overall P300 effect (Chang et al., 2024; Chen et al., 2024; Polich, 2007; Polich and Kok, 1995).

##### 3.2.5.3 Unconscious inhibition subgroup

###### 3.2.5.3.1 Subgroup definition

Restricted to table tennis-specific masking paradigm inhibition tasks, with the core being “automatic response inhibition under low conscious awareness.” The task employs strong masking to deprive consciousness, relying on the basal ganglia-frontal lobe automatic inhibition circuit, and P300 contains P3a components (Polich, 2007; Jaśkowski et al., 2008).

###### 3.2.5.3.2 Typical task analysis

You et al. (You et al., 2018) used a sandwich masking method requiring “non-conscious inhibition of dominant responses”.

###### 3.2.5.3.3 Effect analysis

This subgroup yielded a pooled effect size of SMD = 6.42, 95% CI (5.50, 7.33), *Z* = 13.71, *p* < 0.0001, indicating an association between specialized table tennis training and P300 alterations (Chen et al., 2024; You et al., 2018).

###### 3.2.5.3.4 Heterogeneity and conclusions

Subgroup heterogeneity was *I*^2^ = 0%, indicating high consistency across the two included studies. Crucially, this finding is strictly exploratory due to the extremely small number of studies (*n* = 2) and should not be generalized. Future large-scale, multi-center studies are required to independently validate this extreme effect size. Future studies should subdivide by consciousness level to precisely analyze unconscious inhibitory regulation (You et al., 2018; Jiang et al., 2024).

Differences in effect sizes across subgroups are validated in Figure 6. The test for subgroup differences was statistically significant (*χ*^2^ = 180.71, *df* = 2, *p* < 0.00001, *I*^2^ = 98.9%), confirming that task mechanism is a significant moderator of the relationship between net sports participation and P300 amplitude. Specifically, the conscious spatial cognition subgroup, *SMD* = 1.74, 95% CI (0.86, 2.62), *p* < 0.0001, and unconscious inhibition subgroup, *SMD* = 6.42, 95% CI (5.50, 7.33), *p* < 0.0001, showed significant positive effects, while the action anticipation-response control subgroup, *SMD* = 0.02, 95% CI (−0.23, 0.28), *p* = 0.85, showed no significant effect.

**Figure 6:**
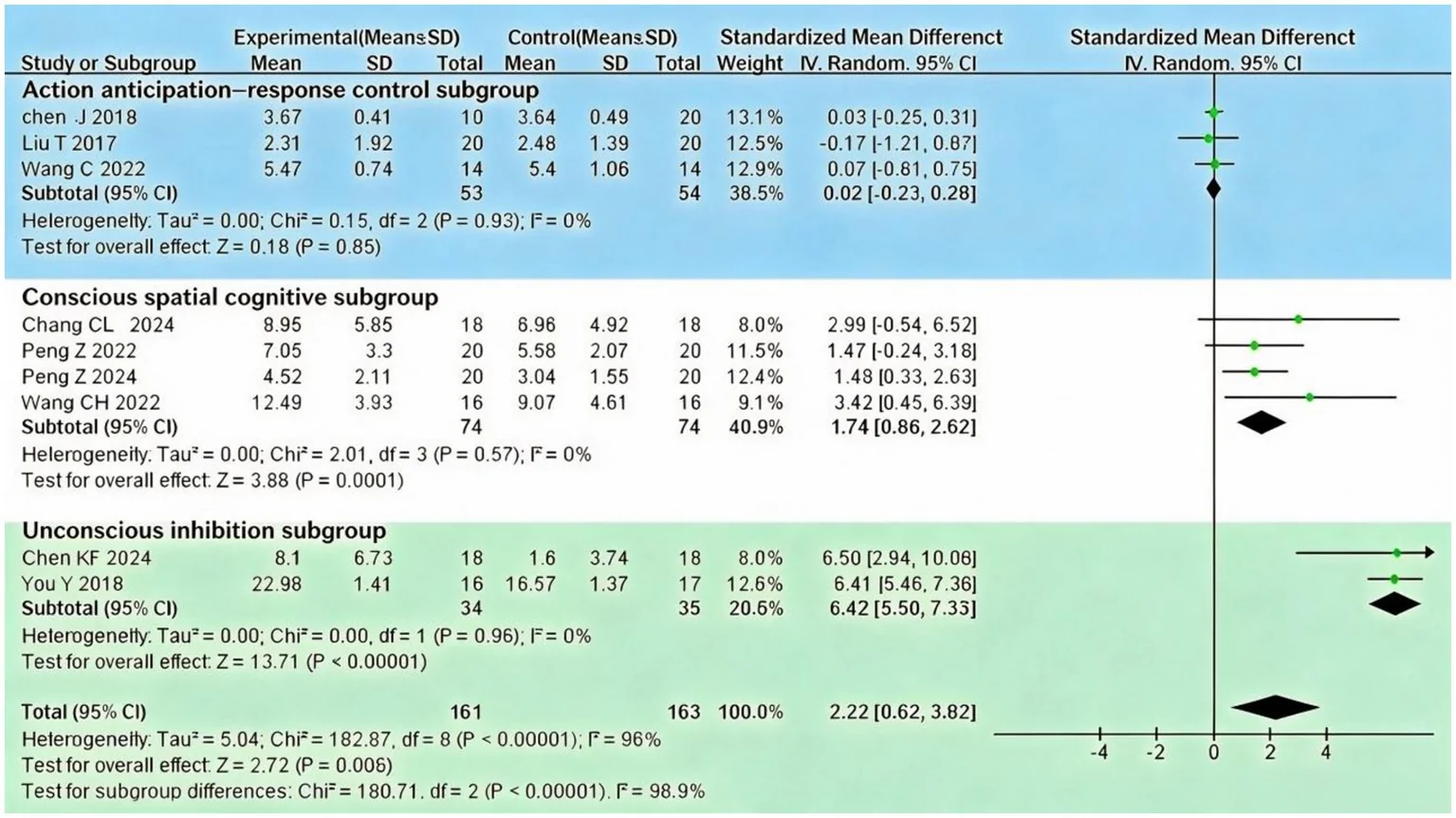
Forest plot comparing P300 amplitude subgroups. The *x*-axis represents the standardized mean difference (*SMD*) of P300 amplitude; horizontal lines represent *95%* confidence intervals (*CIs*); squares indicate individual study weights; diamonds indicate pooled subgroup effect sizes. All statistical tests were two-tailed.

#### 3.2.6 Summary

These subgroup findings provide partial support for both Hypothesis 1 and Hypothesis 2, and reveal a task-dependent dual pattern of P300 modulation. Specifically, the significant amplitude increase in the conscious spatial cognition subgroup aligns with the resource enhancement hypothesis, reflecting strengthened attentional resource capacity in the parietal–frontal network (Polich, 2007; Polich and Kok, 1995). In contrast, the null effect in the action anticipation-response control subgroup is consistent with the neural efficiency hypothesis, indicating that automatized action processing reduces reliance on deliberate attentional allocation (Hommel et al., 2001; Kibele, 2006). The large effect in the unconscious inhibition subgroup represents an exploratory finding that extends beyond our initial *a priori* predictions (Jaśkowski et al., 2008; Jiang et al., 2024).

This divergent pattern is consistent with the neural efficiency hypothesis of expert performance (Hommel et al., 2001; Kibele, 2006). For conscious spatial cognition tasks, long-term net sports training strengthens functional connectivity within the parietal–frontal network, which enhances deliberate attentional allocation and spatial encoding efficiency, reflected in increased P3b amplitude (Polich, 2007; Polich and Kok, 1995). In contrast, action anticipation tasks rely heavily on procedural motor memory; with extensive training, these processes become increasingly automatized and shift from controlled parietal–frontal processing to subcortical motor circuits. This automation reduces the demand for explicit attentional resources, resulting in no net change in P300 amplitude (Hommel et al., 2001; Jiang et al., 2021).

### 3.3 Analysis of the N200 component

#### 3.3.1 Publication bias analysis results

Egger’s regression test showed no statistically significant publication bias for N200 amplitude (*t* = 0.539, *p* = 0.628). However, due to the small number of included studies (*n* = 5), the statistical power of Egger’s test is very low, and this result cannot confirm the absence of publication bias. Publication bias was assessed qualitatively by visual inspection of the funnel plot. The funnel plot’s slight visual asymmetry, Figure 7 was attributed to limited statistical power due to the small sample size (5 studies) (Lewis and Clarke, 2001). Effect differences thus stem from genuine mechanisms rather than publication bias (Egger et al., 1997).

**Figure 7:**
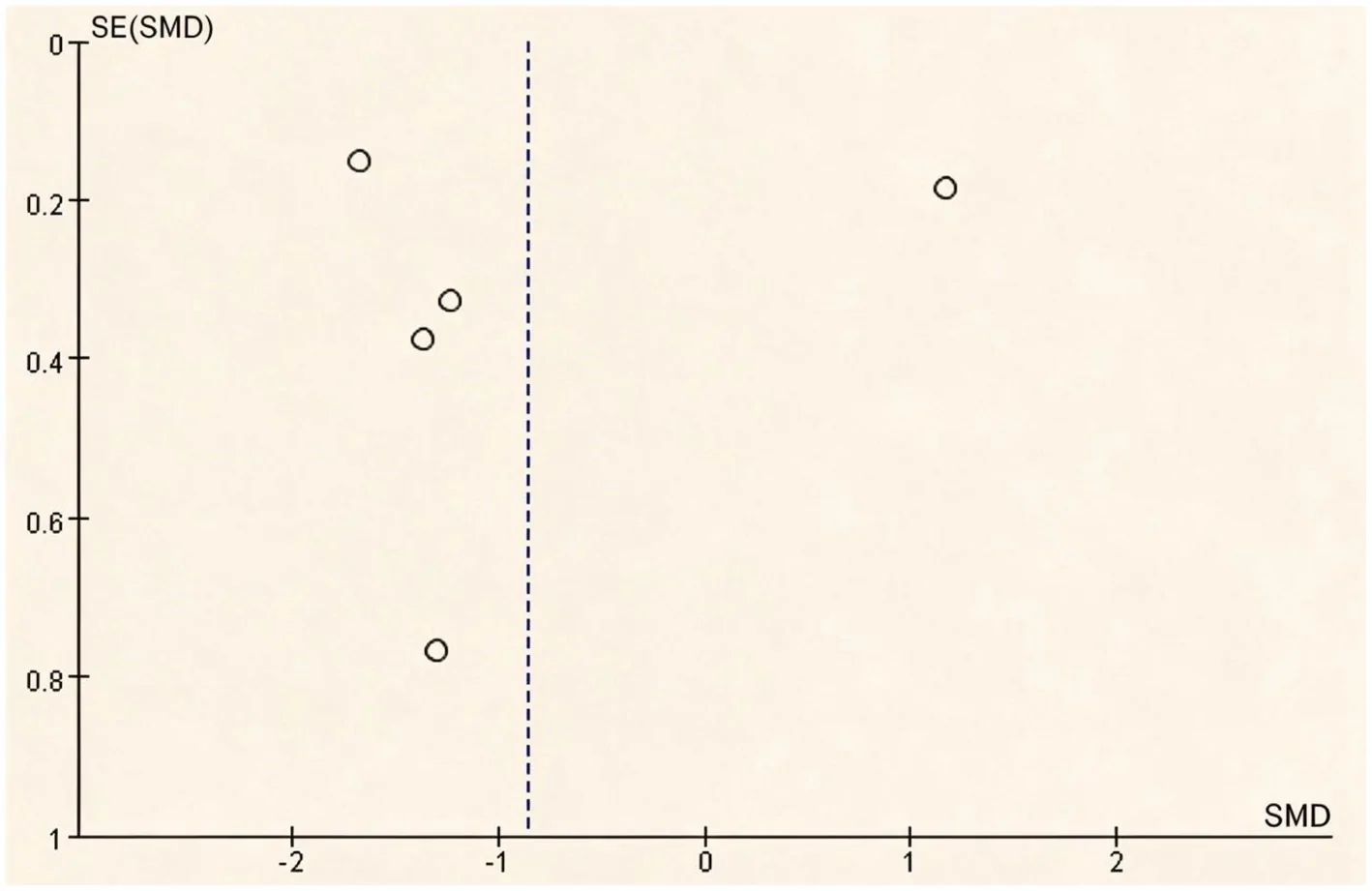
Publication bias funnel plot for N200 amplitude. The *x*-axis represents the standardized mean difference (*SMD*) of N200 amplitude; the *y*-axis represents the standard error of *SMD*. Slight visual asymmetry is due to the small sample size (*n* = 5), with no significant publication bias (Egger’s test, *t = 0*.539, *p* = 0.628 > 0.05). All statistical tests were two-tailed.

#### 3.3.2 Heterogeneity analysis results

The *Q*-test (*χ*^2^ = 149.90, *df* = 4, *p* < 0.00001) revealed extremely high heterogeneity (*I*^2^ = 97%, *Tau*^2^ = 2.46) (Higgins and Thompson, 2002). Core root cause include task mechanism confounding: “conscious response inhibition” (e.g., Chen et al., 2019), and “unconscious automated processing” (e.g., You et al., 2018; Yeung et al., 2004; Kahan and Chokshi, 2013), leading to fluctuations in effect size direction and magnitude (Yeung et al., 2004; Jin et al., 2015).

#### 3.3.3 Effect size results

Figure 8 shows that the overall effect size was *SMD* = −0.86, 95% CI (−2.28, 0.56), *Z* = 1.19, *p* = 0.24, failing to reach statistical significance (Lewis and Clarke, 2001). Conscious response inhibition tasks showed positive effects (*SMD* = 1.17), while conflict monitoring/unconscious processing tasks exhibited negative effects (e.g., Liu et al., 2017, *SMD* = −5.45; Yeung et al., 2004; Jin et al., 2015). This indicates that long-term net sports participation is associated with N200 alterations via distinct task mechanisms, necessitating subgroup analyses (Yeung et al., 2004; Kahan and Chokshi, 2013).

**Figure 8:**
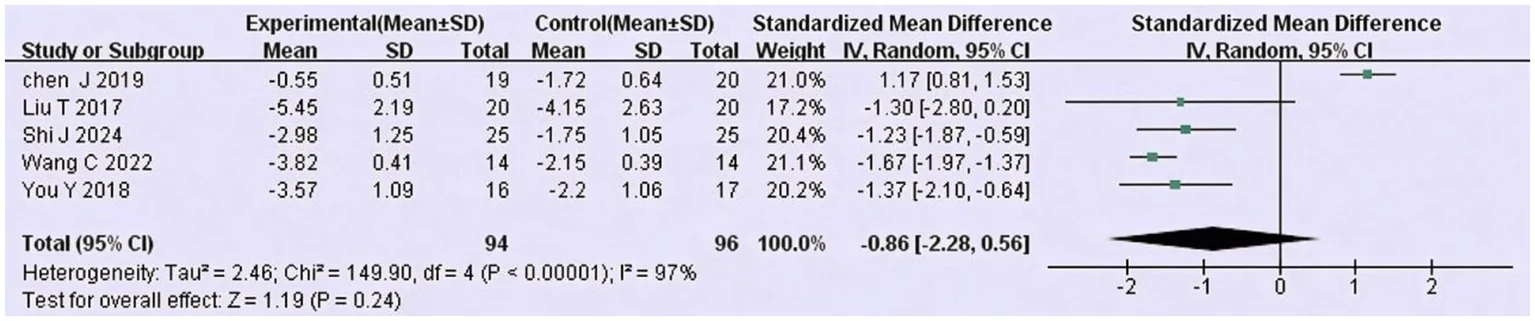
Forest plot comparing N200 amplitudes. The *x*-axis represents the standardized mean difference (*SMD*) of N200 amplitude; horizontal lines represent *95%* confidence intervals (*CIs*); squares represent individual study weights; the diamond represents the pooled effect size. The pooled *SMD* = −0.86, 95% CI (−2.28, 0.56), *p* = 0.24, with extremely high heterogeneity (*I*^2^ = 97%). All statistical tests were two-tailed.

##### 3.3.3.1 Sensitivity analysis by demographic factors

Similarly, stratified sensitivity analyses showed that excluding studies with extreme values of age, gender ratio, or training duration did not significantly alter the pooled effect size (all *p* > 0.05). Formal meta-regression was not performed due to the limited number of included studies, and the moderating role of these demographic factors cannot be definitively concluded.

#### 3.3.4 Sensitivity analysis results

Sensitivity analysis was conducted by sequentially excluding individual studies and using a random-effects model (Higgins et al., 2011). Excluding only Chen et al. (2019) (active response inhibition tasks) reduced heterogeneity from *I*^2^ = 97 to 0% and shifted the pooled effect size to a significant negative value, indicating this study’s unique mechanism as the core source of heterogeneity (Higgins et al., 2011; Yeung et al., 2004). Excluding other studies retained high heterogeneity but a consistent negative effect direction, reflecting training’s stable regulatory effect on passive conflict monitoring/unconscious inhibition tasks (Jin et al., 2015; Kahan and Chokshi, 2013).

#### 3.3.5 Subgroup analysis results

Subgroup analyses were performed post-hoc to explore sources of heterogeneity and were exploratory in nature. Multiple comparisons across subgroups may inflate the Type I error rate, so subgroup results should be interpreted cautiously and not treated as confirmatory conclusions. Sensitivity analysis identified Chen et al. (2019) as the primary source of N200 heterogeneity. To test Hypothesis 3 regarding bidirectional, task-dependent N200 modulation, we conducted subgroup analysis stratified by cognitive control mode and underlying neural circuits.

Based on N200’s core functions, conscious engagement, and neural circuit differences, N200 responses were categorized into three subgroups (Yeung et al., 2004; Kahan and Chokshi, 2013). Publication bias testing, Figure 9 showed no significant asymmetry across subgroups, validating that effect differences stem from genuine mechanisms (Egger et al., 1997). No statistically significant within-subgroup heterogeneity was detected after subgroup stratification. However, given the small number of studies per subgroup, the *I*^2^ statistic has low statistical power, and this result should be interpreted with caution. Specific analyses are as follows:

**Figure 9:**
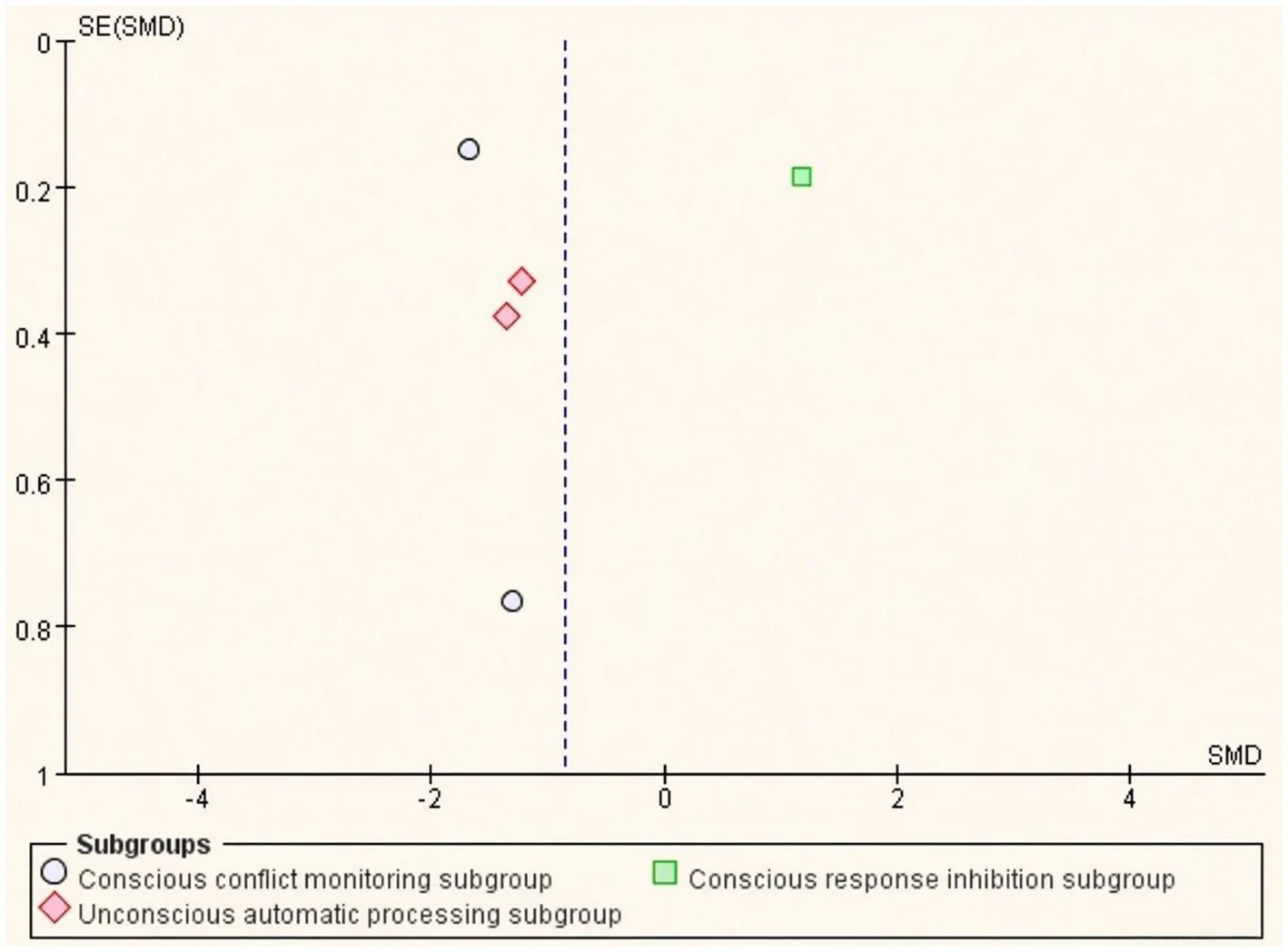
Publication bias funnel plot for N200 amplitude subgroups. The *x*-axis represents the standardized mean difference (*SMD*) of N200 amplitude; the *y*-axis represents the standard error of *SMD*. Three subgroups: Conscious conflict monitoring, unconscious automatic processing, and conscious response inhibition. All funnels show no significant asymmetry (Egger’s test, *p* > 0.05). All statistical tests were two-tailed.

##### 3.3.5.1 Conscious conflict monitoring subgroup

###### 3.3.5.1.1 Subgroup definition

Covers non-inhibitory conscious conflict detection tasks that rely on the prefrontal-anterior cingulate network. N200 amplitude changes reflect regulation of active conflict detection efficiency (Yeung et al., 2004; Kahan and Chokshi, 2013).

###### 3.3.5.1.2 Representative task analysis

Includes badminton action conflict anticipation detection and tennis deep conflict recognition.

###### 3.3.5.1.3 Effect analysis

The subgroup exhibits a significantly negative effect size, *SMD* = −1.66, 95% CI (−1.95, −1.37), *Z* = 11.16, *p* < 0.00001, indicating that long-term net sports participation is associated with significant negative alterations in N200 amplitude. Reduced N200 amplitude is associated with enhanced neural efficiency in early conflict detection (Yeung et al., 2004; Huang et al., 2024).

###### 3.3.5.1.4 Heterogeneity and conclusions

No statistically significant within-subgroup heterogeneity was detected (*I*^2^ = 0%). However, the statistical power of the *I*^2^ statistic is limited when the number of studies is small, and this result cannot confirm mechanistic purity. Repeated exposure to sport-specific dynamic conflict stimuli enhances the efficiency of early conflict detection: the anterior cingulate cortex identifies conflicting information faster and with lower metabolic resource consumption, which is electrophysiologically reflected as reduced N200 amplitude. This optimized early processing also lays a more efficient neural foundation for subsequent P300-mediated late-stage decision-making (Yeung et al., 2004; Kahan and Chokshi, 2013).

##### 3.3.5.2 Unconscious automatic processing subgroup

###### 3.3.5.2.1 Subgroup definition

Limited to masked-paradigm unconscious conflict resolution tasks that rely on the occipital-parietal network’s automatic inhibitory mechanism (Jaśkowski et al., 2008; Kahan and Chokshi, 2013).

###### 3.3.5.2.2 Representative task analysis

Includes masked inhibition tasks with ping-pong ball stimuli and masked activation conflicts.

###### 3.3.5.2.3 Effect analysis

The subgroup showed a significantly negative effect size, *SMD* = −1.29, 95% CI (−1.77, −0.81), *Z* = 5.24, *p* < 0.00001, indicating negative alterations in N200 amplitude. Reduced N200 amplitude is associated with enhanced neural economy in unconscious conflict resolution (Jaśkowski et al., 2008; Jiang et al., 2024).

###### 3.3.5.2.4 Heterogeneity and conclusions

Subgroup heterogeneity was 0%, supporting the idea that motor training may be associated with improved neuroeconomy of unconscious processing (You et al., 2018; Kahan and Chokshi, 2013).

##### 3.3.5.3 Conscious response inhibition subgroup

###### 3.3.5.3.1 Subgroup definition

Limited to active behavioral inhibition tasks that rely on the frontal lobe-basal ganglia circuit. N200 amplitude changes reflect regulation of active inhibition resource allocation (Yeung et al., 2004; Kahan and Chokshi, 2013).

###### 3.3.5.3.2 Typical task analysis

Badminton Reaction Suppression, distinct from other conflict monitoring tasks (Yeung et al., 2004).

###### 3.3.5.3.3 Effect analysis

This subgroup only Chen et al. (2019) showed a significantly positive effect size, *SMD* = 1.17, 95% CI (0.81, 1.53), *Z* = 6.33, *p* < 0.00001, indicating positive alterations in N200 amplitude. Increased amplitude reflects additional neural resources required for active inhibition (Yeung et al., 2004; Kahan and Chokshi, 2013).

###### 3.3.5.3.4 Heterogeneity and conclusions

No heterogeneity analysis was applicable as only one study was included in this subgroup. This result is preliminary and strictly exploratory. Mechanistically, active response inhibition relies on the frontal-basal ganglia inhibitory control circuit: voluntary suppression of prepotent motor responses requires active recruitment of prefrontal cortex and subthalamic nucleus resources. For net sport athletes, long-term training refines this inhibitory control pathway, enabling greater mobilization of neural resources during high-demand stop-signal tasks, which manifests as increased N200 amplitude. This pattern is diametrically opposed to the efficiency optimization seen in passive conflict monitoring tasks, constituting the core source of overall N200 heterogeneity (Yeung et al., 2004; Jin et al., 2015).

This mechanism opposition is statistically confirmed by the test for subgroup differences (*χ*^2^ = 149.60, *df* = 2, *p* < 0.00001, *I*^2^ = 88.7%), which indicates that task mechanism explains 88.7% of the between-study heterogeneity in N200 effects. As shown in Figure 10, the conscious conflict monitoring subgroup, *SMD* = −1.66, 95% CI (−1.95, −1.37), *p* < 0.00001, and unconscious automatic processing subgroup, *SMD* = −1.29, 95% CI (−1.77, −0.81), *p* < 0.00001, showed significant negative effects, while the conscious response inhibition subgroup, *SMD* = 1.17, 95% CI (0.81, 1.53), *p* < 0.00001, showed a significant positive effect. Non-overlapping 95% CIs further validate that task mechanism is the primary determinant of N200 modulation direction.

**Figure 10:**
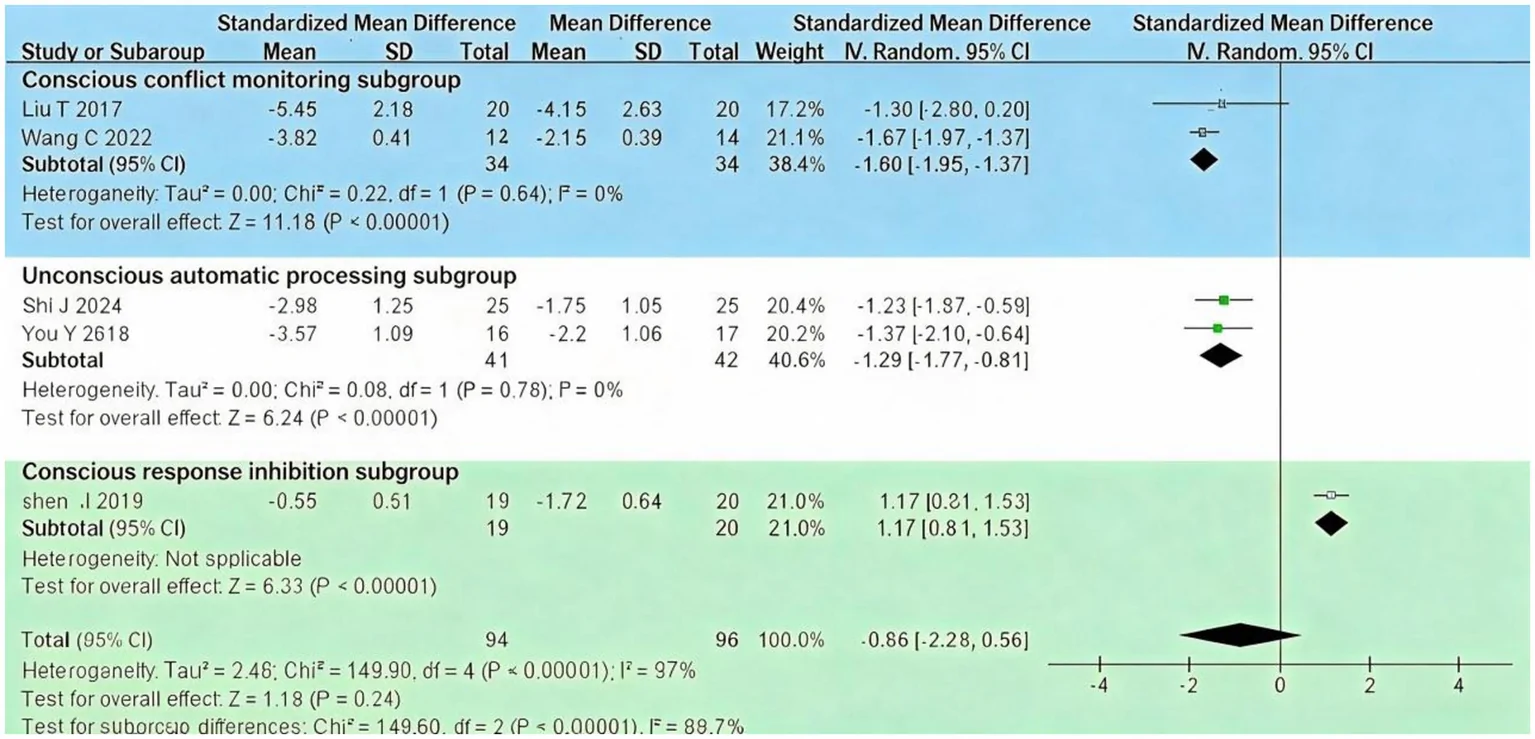
Forest plot comparing N200 amplitude subgroups. The *x*-axis represents the standardized mean difference (*SMD*) of N200 amplitude; horizontal lines represent 95% confidence intervals (*CIs*); squares represent individual study weights; diamonds represent subgroup pooled effect sizes. Subgroup results: Conscious conflict monitoring (*SMD* = −1.66, *p* < 0.00001, *I*^2^ = 0%), unconscious automatic processing (*SMD* = −1.29, *p* < 0.00001, *I*^2^ = 0%), and conscious response inhibition (*SMD* = 1.17, *p* < 0.00001). All statistical tests were two-tailed.

#### 3.3.6 Summary

These bidirectional modulation patterns fully support Hypothesis 3: N200 amplitude changes are determined by the type of cognitive control engaged. Reduced amplitudes in passive conflict monitoring and automatic processing tasks align with the neural efficiency hypothesis, reflecting optimized early conflict detection in the prefrontal-anterior cingulate circuit (Yeung et al., 2004; Kahan and Chokshi, 2013). In contrast, increased amplitudes in active response inhibition tasks reflect greater recruitment of inhibitory control resources in the frontal-basal ganglia circuit, consistent with a resource allocation account (Yeung et al., 2004; Jin et al., 2015). The overall effect was non-significant (*SMD* = −0.86, *p* = 0.24) (Lewis and Clarke, 2001). Sensitivity analysis confirmed consistent results across studies excluding Chen et al. (2019), Higgins et al. (2011), and Jin et al. (2015).

This bidirectional modulation of N200 amplitude reflects two dissociable cognitive control modes supported by distinct neural circuits (Yeung et al., 2004; Folstein and Van Petten, 2008). Reduced N200 amplitude in passive conflict monitoring and automatic processing tasks indicates enhanced neural efficiency: long-term training optimizes processing in the prefrontal-anterior cingulate and occipital-parietal networks, enabling faster, more resource-efficient early conflict detection (Huang et al., 2024; Kahan and Chokshi, 2013). In contrast, increased N200 amplitude in active response inhibition tasks reflects greater recruitment of neural resources in the frontal-basal ganglia circuit, which is required for the voluntary suppression of prepotent motor responses (Yeung et al., 2004; Jin et al., 2015).

## 4 Discussion

### 4.1 Key innovations and theoretical and practical contributions

This meta-analysis possesses prominent methodological, empirical, and translational innovations compared with previous evidence syntheses in exercise neuroscience.

#### 4.1.1 Theoretical and framework contribution

The primary theoretical contribution of this meta-analysis is to help reconcile the long-standing contradiction between the neural efficiency hypothesis and the resource enhancement hypothesis in the context of open-skill sport expertise. Prior syntheses have produced conflicting findings by pooling across heterogeneous tasks, leading to an apparent paradox: some studies report larger ERP amplitudes in athletes, while others report reduced amplitudes.

By adopting a mechanism-driven subgroup framework stratified by cognitive function, conscious processing level, and neural circuit, we demonstrate that the two competing hypotheses are not mutually exclusive-instead, they describe distinct modes of sport-induced neuroplasticity that are task-dependent. Specifically, tasks requiring deliberate top-down control (e.g., spatial cognition) engage resource enhancement mechanisms, whereas tasks relying on automatized processing (e.g., action anticipation) engage neural efficiency mechanisms. This task-dependent framework unifies disparate empirical findings and provides a coherent theoretical account of experience-dependent neuroplasticity in net sports, aligning with established cognitive theories of ERP generation (Polich, 2007; Yeung et al., 2004; Folstein and Van Petten, 2008).

#### 4.1.2 Empirical breakthrough on ERP modulation patterns

Empirically, this study provides the first meta-analytic confirmation of task-specific, bidirectional ERP modulation in net sport athletes, corresponding directly to our three *a priori* hypotheses. For P300, we replicate a robust enhancement effect in conscious spatial cognition tasks and a null effect in automatized action tasks, supporting the dual-mechanism account. For N200, we systematically demonstrate bidirectional modulation: reduced amplitudes in passive conflict monitoring and automatic processing, and increased amplitudes in active response inhibition. This dissociation explains the extreme between-study heterogeneity reported in prior literature and provides a unified electrophysiological framework for understanding decades of inconsistent findings in sport ERP research (Jiang et al., 2021). The relatively large effect sizes observed in this study are primarily attributed to sport-specific cognitive paradigms rather than general cognitive tasks. Domain-specific paradigms maximize the detection of expertise-related neurocognitive differences, resulting in larger effect sizes than generic paradigms such as the digit Stroop task.

#### 4.1.3 Methodological rigor improvement

Considering the characteristics of included cross-sectional studies and notable publication bias in P300-related literature, we adopted a full set of bias detection and sensitivity strategies. We strictly distinguished correlational associations from causal inferences and explicitly interpreted the limitations of observational data. Meanwhile, we summarized common methodological defects in current sports ERP experiments and put forward targeted suggestions for standardized ERP recording in future primary studies.

#### 4.1.4 Translational value for sports practice

The observed task-specific ERP patterns provide candidate electrophysiological indicators and a theoretical basis for developing differentiated cognitive training paradigms for badminton and table tennis. However, these training implications remain hypothetical at this stage, as cross-sectional evidence cannot confirm the intervention effects of such training. Future randomized controlled trials are needed to test their effectiveness. This approach aligns with the framework of precision motor-cognitive training proposed in recent sport neuroscience reviews (Jiang et al., 2021; Jiang et al., 2024). It also provides feasible indicators for long-term cognitive state monitoring of elite athletes.

#### 4.1.5 Prospects for follow-up research

We systematically summarized the overall limitations of current literature, including single demographic distribution, insufficient longitudinal intervention evidence, and small sample sizes in partial subgroups, and pointed out clear directions for subsequent exploration.

#### 4.1.6 Neural circuit mechanisms of task-dependent modulation

Notably, the neural circuit interpretations presented in this section are theoretical inferences derived from existing cognitive neuroscience literature. The present study only measured scalp ERP amplitudes and did not directly verify the activity of specific brain circuits. Future neuroimaging studies (e.g., fMRI and ERP source localization) are needed to validate these hypothetical mechanisms.

Taken together, the task-specific modulation of N200 and P300 observed in this meta-analysis can be mapped to two dissociable neuroplasticity pathways, corresponding to the two competing theoretical frameworks.

For tasks requiring deliberate, top-down cognitive control (conscious spatial cognition and active response inhibition), the observed amplitude modulation patterns suggest that net sport training may be associated with resource enhancement-type plasticity in cortical control networks. Specifically, spatial cognition tasks engage the parietal–frontal attention network, with training strengthening inter-regional functional connectivity and expanding attentional resource capacity, reflected in increased P3b amplitude. Active response inhibition tasks engage the frontal-basal ganglia inhibitory circuit, with training enhancing the ability to recruit inhibitory control resources, reflected in increased N200 amplitude.

For tasks relying on automatic or passive processing (action anticipation, passive conflict monitoring, and unconscious processing), training may be associated with neural efficiency-type plasticity, characterized by reduced cortical resource consumption. Action anticipation tasks shift from cortical parietal–frontal processing to automatic subcortical motor circuits, resulting in no net change in P300 amplitude. Passive conflict monitoring and unconscious processing tasks optimize processing efficiency in the prefrontal-anterior cingulate and occipital-parietal networks, respectively, reducing N200 amplitude.

This circuit-specific dual-pathway model provides a unified neurobiological account for the heterogeneous ERP findings in prior sport neuroscience literature, and demonstrates that experience-dependent neuroplasticity is not a global, uniform phenomenon, but is shaped by the specific cognitive demands and corresponding neural circuits of the task.

#### 4.1.7 Evidence quality assessment

The overall evidence quality was evaluated using the Grading of Recommendations Assessment, Development and Evaluation (GRADE) framework, which rates evidence quality as high, moderate, low, or very low based on study limitations, inconsistency, indirectness, imprecision, and publication bias.

Overall evidence for P300 modulation: Rated as moderate quality. Downgraded one level due to significant publication bias (Egger’s test, *p* = 0.010) and moderate methodological heterogeneity across included studies (0 points on the NOS comparability domain). Upgraded one level for the large magnitude of effect (*SMD* = 2.22) and consistent results in sensitivity analysis.

Overall evidence for N200 modulation: Rated as low quality. Downgraded two levels due to the small number of eligible studies (*n* = 5) and extreme between-study heterogeneity (*I*^2^ = 97%) that could not be fully explained by subgroup analysis.

Subgroup evidence quality:

Conscious spatial cognition subgroup (P300): Moderate quality (consistent results and no publication bias).

Unconscious inhibition subgroup (P300): Low quality (only two studies, exploratory finding).

Conscious conflict monitoring subgroup (N200): Low quality (only two studies).

Conscious response inhibition subgroup (N200): Very low quality (only one study, preliminary finding).

### 4.2 Main conclusions

This meta-analysis evaluated the regulatory patterns of N200 and P300 components in ERP following net sports training (badminton, table tennis, and tennis) (Wang et al., 2022; Chen et al., 2024). Through multiple analyses and mechanism-specific subgroupings, the following conclusions were drawn:

#### 4.2.1 Task mechanism specificity in P300 modulation

Overall, a significant positive effect was observed (*SMD* = 2.22, *p* = 0.006) (Lewis and Clarke, 2001). Due to high heterogeneity (*I*^2^ = 96%) (Higgins and Thompson, 2002) and positive publication bias (Egger et al., 1997), subgroup analysis was necessary. Results showed: long-term net sports participation was significantly associated with increased P300 amplitude in conscious spatial cognition tasks, which may be associated with potential functional plasticity of the parietal–frontal network (Polich, 2007; Polich and Kok, 1995); no significant effect was observed in action anticipation-response control tasks (*SMD* = 0.02, *p* = 0.85) (Hommel et al., 2001; Kibele, 2006); table tennis-specific unconscious inhibition tasks showed a statistically significant large effect size (*SMD* = 6.42, *p* < 0.0001). This finding is preliminary and requires independent validation in large-scale studies.

#### 4.2.2 N200 regulation varies with conflict processing type

The overall effect was non-significant (*SMD* = −0.86, *p* = 0.24) (Lewis and Clarke, 2001), stemming from high task mechanism heterogeneity (*I*^2^ = 97%) (Higgins and Thompson, 2002) rather than publication bias (Egger et al., 1997). Subgroup analyses revealed that conscious conflict monitoring and unconscious automatic processing tasks reduced N200 amplitude via enhanced neural efficiency (Yeung et al., 2004; Kahan and Chokshi, 2013); conscious response inhibition tasks increased amplitude due to heightened resource allocation (Yeung et al., 2004; Jin et al., 2015). This opposition constitutes the core source of heterogeneity (Yeung et al., 2004).

#### 4.2.3 Key modulating factors for effect differentiation

P300 effect differences correlate with “conscious spatial coding vs. action automation processing” (Polich, 2007; Polich and Kok, 1995), while N200 differentiation is driven by neural circuitry variations (Yeung et al., 2004; Kahan and Chokshi, 2013). Sensitivity analyses validated the robustness of both component effects (Higgins et al., 2011).

Theoretically, this study provides a unified framework for understanding the effects of net-based sports on cognitive function, demonstrating that ERP component changes are not uniform but depend on the specific cognitive demands of the task. This challenges the traditional view that exercise has a general enhancing effect on cognitive function and highlights the importance of task specificity in exercise neuroscience research.

Practically, this research provides evidence for “motor-cognitive” neuroplasticity (Huang et al., 2024; Jin et al., 2015) and offers preliminary guidance for specialized training (Jiang et al., 2021; Jiang et al., 2024). These findings provide preliminary implications for future research: P300 amplitude in spatial cognition tasks and N200 amplitude in inhibition tasks may serve as candidate electrophysiological indicators for monitoring athletes’ cognitive status. However, their predictive validity, test–retest reliability, and practical value for training assessment need to be verified in future longitudinal and intervention studies.

Collectively, these findings support a task-dependent dual-mechanism framework of sport-induced neuroplasticity that reconciles the neural efficiency and resource enhancement hypotheses. P300 enhancement is observed primarily in tasks requiring conscious spatial encoding, whereas automatized action tasks show no net amplitude change, reflecting efficiency gains. N200 modulation follows a similar task-specific pattern, with reduced amplitudes in passive monitoring and increased amplitudes in active inhibition. This framework provides a unified theoretical account of heterogeneous prior findings and offers electrophysiological guidance for designing precise, mechanism-targeted motor-cognitive training programs.

## 5 Limitations

Several limitations should be considered when interpreting these findings.

First, all included studies adopted an observational cross-sectional expert-novice design, with only one quasi-experimental intervention study. This level of evidence can only reveal the association between net sport experience and ERP components, and cannot confirm a causal relationship between long-term training and neuroplastic changes. Future randomized controlled trials are required to verify causal effects.

Second, only English-language studies were included, which may introduce language bias. Although we searched the Chinese database CNKI, no Chinese-language studies met all inclusion criteria due to incomplete ERP data reporting (e.g., missing standard deviations of amplitude values). Future meta-analyses should include non-English literature to improve generalizability.

Third, variability in ERP recording protocols across studies (e.g., electrode arrays, filtering ranges, and component time window definitions) may have introduced methodological heterogeneity and affected effect size stability. This is a potential source of residual heterogeneity, and standardized ERP recording protocols are needed in future primary research.

Fourth, significant publication bias was detected for P300 (Egger’s test, *t* = −3.48, *p* = 0.010), which may have overestimated the positive effect. Inclusion of gray literature will be important for verifying the robustness of this result.

Fifth, most included studies focused on young men (18–28 years old), with limited diversity in gender and age. This restricts the generalizability of findings to female athletes and other age groups.

Sixth, the number of studies in several subgroups is small (e.g., only one study in the N200 conscious response inhibition subgroup), which reduces the statistical power and robustness of subgroup-specific conclusions. These exploratory findings require independent validation in larger study sets.

Seventh, due to the limited total number of included studies, formal meta-regression analysis could not be performed to simultaneously control for multiple confounding factors. Future meta-analyses with a larger evidence base should use meta-regression to further explore independent sources of heterogeneity.

Eighth, subgroup analysis by specific sport type (badminton vs. table tennis vs. tennis) was not performed due to the small number of studies per sport. Whether the modulation patterns of N200 and P300 components differ across net sports remains to be explored in future studies with a larger evidence base.

Future studies should expand sample sizes (Egger et al., 1997), refine subgroups (Higgins et al., 2011), and correct for publication bias (Egger et al., 1997) to refine effect patterns.

## Data Availability

The raw data supporting the conclusions of this article will be made available by the corresponding author upon reasonable request.

## References

[ref1] Chang C. L. Gan Y. C. Pan C. Y. Tseng Y. T. Wang T. C. Tsai C. L. (2024). Neurocognitive performance of badminton players at different competitive levels in visuospatial attention tasks. Exp. Brain Res. 242, 1933–1946. doi: 10.1007/s00221-024-06872-1, 38900160

[ref2] Chen K. F. Chueh T. Y. Hung T. M. (2024). Differences in visuospatial cognition among table tennis players of different skill levels: An event-related potential study. PeerJ 12:e17295. doi: 10.7717/peerj.17295, 38827290 PMC11144390

[ref3] Chen J. Li Y. Zhang G. Jin X. Lu Y. Zhou C. (2019). Enhanced inhibitory control during re-engagement processing in badminton athletes: An event-related potential study. J. Sport Health Sci. 8, 585–594. doi: 10.1016/j.jshs.2019.05.005, 31720072 PMC6834996

[ref4] Cohen J. D. Botvinick M. Carter C. S. (2000). Anterior cingulate and prefrontal cortex: who’s in control? Nat. Neurosci. 3, 421–423. doi: 10.1038/74783, 10769376

[ref5] Egger M. Smith G. D. Schneider M. Minder C. (1997). Bias in meta-analysis detected by a simple, graphical test. BMJ 315, 629–634. doi: 10.1136/bmj.315.7109.629, 9310563 PMC2127453

[ref6] Folstein J. R. Van Petten C. (2008). Influence of cognitive control and mismatch on the N2 component of the ERP: a review. Psychophysiology 45, 152–170. doi: 10.1111/j.1469-8986.2007.00602.x, 17850238 PMC2365910

[ref7] Heinemann A. Kiesel A. Pohl C. Kunde W. (2010). Masked response priming in expert typists. Conscious. Cogn. 19, 399–407. doi: 10.1016/j.concog.2009.09.003 19819732

[ref8] Higgins J. P. Altman D. G. Gøtzsche P. C. Jüni P. Moher D. Oxman A. D. . (2011). The Cochrane collaboration's tool for assessing risk of bias in randomised trials. BMJ 343:d5928. doi: 10.1136/bmj.d5928 PMC3196245 22008217

[ref9] Higgins J. P. Thompson S. G. (2002). Quantifying heterogeneity in a meta-analysis. Stat. Med. 21, 1539–1558. doi: 10.1002/sim.1186, 12111919

[ref10] Hommel B. Müsseler J. Aschersleben G. Prinz W. (2001). The theory of event coding (TEC): a framework for perception and action planning. Behav. Brain Sci. 24, 849–878. doi: 10.1017/S0140525X01000103, 12239891

[ref11] Huang X. Wong B. W. L. Ng H. T. Sommer W. Dimigen O. Maurer U. (2024). Neural mechanisms underlying preview effects and masked priming effects in visual word processing. Atten. Percept. Psychophys. 87, 5–24. doi: 10.3758/s13414-024-02904-8 PMC11845427 38956004

[ref12] Jaśkowski P. Białunska A. Tomanek M. Verleger R. (2008). Mask and distractor triggered inhibitory processes in the priming of motor responses: an EEG study. Psychophysiology 45, 70–85. doi: 10.1111/j.1469-8986.2007.00595.x, 17887961

[ref13] Jiang R. Xie F. Li A. (2021). Motor expertise and performance in sport-specific priming tasks: a systematic review and meta-analysis. PeerJ 9:e11243. doi: 10.7717/peerj.11243, 33954051 PMC8051356

[ref14] Jiang R. Xie F. Li A. (2024). Effect of conscious conflict on the subliminal perception of table tennis players: from the electrophysiological evidence of ERP. Cogn. Neurodyn. 18, 85–94. doi: 10.1007/s11571-022-09883-2, 38406196 PMC10881928

[ref15] Jin C. Li A. Tao Y. (2015). The advantage of identifying dynamic image information of table tennis players: an event-related potentials study. Chin. J. Sports Med. 34, 181–187. doi: 10.16038/j.1000-6710.2015.02.014

[ref16] Kahan T. A. Chokshi N. S. (2013). Perceptual interactions between primes, masks, and targets: further evidence for object updating. Vis. Cogn. 21, 123–138. doi: 10.1080/13506285.2013.788112

[ref17] Kibele A. (2006). Non-consciously controlled decision making for fast motor reactions in sports—a priming approach for motor responses to non-consciously perceived movement features. Psychol. Sport Exerc. 7, 591–610. doi: 10.1016/j.psychsport.2006.05.001

[ref18] Kiefer M. Martens U. (2010). Attentional sensitization of unconscious cognition: task sets modulate subsequent masked semantic priming. J. Exp. Psychol. Gen. 139, 464–489. doi: 10.1037/a0019561 20677895

[ref19] Lewis S. Clarke M. (2001). Forest plots: trying to see the wood and the trees. BMJ 322, 1479–1480. doi: 10.1136/bmj.322.7300.1479, 11408310 PMC1120528

[ref20] Liu T. Shao M. Yin D. Li Y. Yang N. Yin R. . (2017). The effect of badminton training on the ability of same-domain action anticipation for adult novices: evidence from behavior and ERPs. Neurosci. Lett. 660, 6–11. doi: 10.1016/j.neulet.2017.08.038, 28830821

[ref21] MacDonald A. W. Cohen J. D. Stenger V. A. Carter C. S. (2000). Dissociating the role of the dorsolateral prefrontal and anterior cingulate cortex in cognitive control. Science 288, 1835–1838. doi: 10.1126/science.288.5472.1835, 10846167

[ref22] Patel A. D. Azzam P. N. (2005). Event-related potentials in cognitive neuroscience: an introduction. J. Clin. Neurophysiol. 22, 1–9. doi: 10.7150/ijms.2.147

[ref23] Peng Z. Xu L. Lian J. An X. Chen S. Shao Y. . (2024). Perceptual information processing in table tennis players: based on top-down hierarchical predictive coding. Cogn. Neurodyn. 18, 3951–3961. doi: 10.1007/s11571-024-10171-4, 39712138 PMC11655933

[ref24] Peng Z. Xu L. Wang H. Song T. Shao Y. Liu Q. . (2022). The lateralization of spatial cognition in table tennis players: neuroplasticity in the dominant hemisphere. Brain Sci. 12:1607. doi: 10.3390/brainsci12121607, 36552067 PMC9775476

[ref25] Polich J. (2007). Updating P300: An integrative theory of P3a and P3b. Clin. Neurophysiol. 118, 2128–2148. doi: 10.1016/j.clinph.2007.04.019, 17573239 PMC2715154

[ref26] Polich J. Kok A. (1995). Cognitive and biological determinants of P300: an integrative review. Biol. Psychol. 41, 103–146. doi: 10.1016/0301-0511(95)05130-9, 8534788

[ref27] Shi J. Huang H. Nasrallah F. A. Li A. (2024). High neural efficiency in unconscious perceptual processing among table tennis athletes: An event-related potential study. Brain Sci. 14:756. doi: 10.3390/brainsci14080756, 39199451 PMC11352455

[ref28] Sweller J. (1988). Cognitive load during problem solving: effects on learning. Cogn. Sci. 12, 257–285. doi: 10.1207/s15516709cog1202_4

[ref29] Vogel E. K. McCollough A. W. Machizawa M. G. (2005). Neural measures reveal individual differences in controlling access to working memory. Nature 438, 500–503. doi: 10.1038/nature04171, 16306992

[ref30] Wang C. H. Tu K. C. (2017). Neural correlates of expert behavior during a domain-specific attentional cueing task in badminton players. J. Sport Exerc. Psychol. 39, 209–221. doi: 10.1123/jsep.2016-0335, 28891736

[ref31] Wang C. Yan A. Deng W. Qi C. (2022). Effect of tennis expertise on motion-in-depth perception at different speeds: An event-related potential study. Brain Sci. 12:1160. doi: 10.3390/brainsci12091160, 36138895 PMC9496960

[ref32] Yeung N. Botvinick M. M. Cohen J. D. (2004). The neural basis of error detection: conflict monitoring and the error-related negativity. Psychol. Rev. 111, 931–959. doi: 10.1037/0033-295X.111.4.931, 15482068

[ref33] You Y. Ma Y. Ji Z. Meng F. Li A. Zhang C. (2018). Unconscious response inhibition differences between table tennis athletes and non-athletes. PeerJ 6:e5548. doi: 10.7717/peerj.5548, 30210943 PMC6130236

