# Supplementary material for: Meta-analysis of task- and mechanism-dependent effects of net sports on N200 and P300 components
Source: Front Psychol. 2026 Sep 25;17:1903090. doi: 10.3389/fpsyg.2026.1903090 (PMC13649459; doi:10.3389/fpsyg.2026.1903090)
Supplement: Supplementary file 1 [file Table_1.docx]

Supplementary A: PRISMA 2020 Checklist. This checklist details the compliance of this meta-analysis with PRISMA 2020 guidelines, including literature search, data extraction, risk of bias assessment, and result reporting. Each item corresponds to the reporting requirements specified in the PRISMA 2020 statement, with 'Yes' indicating full compliance, 'Partial' indicating partial compliance, and 'Not applicable' indicating the item is irrelevant to this study.

| **Section and Topic** | **Item #** | **Checklist item** | **Location where item is reported** |
| --- | --- | --- | --- |
| **TITLE** | | |  |
| Title | 1 | Meta-Analysis of the Effects of Net Sports on N200 and P300 Components. | Manuscript home page title section |
| **ABSTRACT** | | |  |
| Abstract | 2 | See the PRISMA 2020 for Abstracts checklist. | The abstract section of the manuscript (which includes objectives, methods, results, and conclusions, complying with the PRISMA 2020 guidelines: Objective: To systematically evaluate the moderating effects of netted movement on ERP N200/P300; Methods: A total of 10 studies (9 cross-sectional studies and 1 quasi-experimental study) from 2016 to 2025 were reviewed, with random effect model meta-analysis and subgroup analysis for heterogeneity exploration; Results: Overall positive effect observed in P300, no overall effect in N200, with significant subgroup differences; Conclusion: The moderating effect of netted movement is task-mechanism specific) |
| **INTRODUCTION** | | |  |
| Rationale | 3 | Describe the rationale for the review in the context of existing knowledge. | 0 Introduction Section (Background: The ERP N200 (stimulus-related categorization and conflict monitoring) and P300 (attention allocation and decision-making) serve as key neurophysiological markers of cognitive processing. While the cognitive shaping effects of specialized training in racquet sports (badminton, table tennis, tennis) have garnered attention, existing research shows inconsistent conclusions regarding the regulatory mechanisms of N200/P300 responses. The heterogeneity in these findings remains unclear, necessitating this meta-analysis to clarify the underlying causes.) |
| Objectives | 4 | Provide an explicit statement of the objective(s) or question(s) the review addresses. | 0 Introduction and Abstract Section (Research Objectives: 1. To systematically evaluate the regulatory patterns of N200 and P300 components in ERP following netted exercise training; 2. To clarify the specificity of training effects (e.g., differences between various exercise types and task categories); 3. To elucidate the sources of effect heterogeneity (e.g., task mechanisms, levels of consciousness, neural circuits) |
| **METHODS** | | |  |
| Eligibility criteria | 5 | Specify the inclusion and exclusion criteria for the review and how studies were grouped for the syntheses. | 1.2 Inclusion/Exclusion Criteria Section (1) Inclusion Criteria: ① Only ERP studies on "scepticis playing with a racket (badminton/tennis/table tennis)" are included, excluding non-net sports; ② Studies must contain at least one specialized motor cognition task, excluding those with only general cognitive tasks; ③ Reports must include raw waveform amplitudes (μV), latency times, and statistical parameters for P300/N200 waves, excluding purely qualitative descriptions; ④ Sample size ≥10 (given the small sample sizes in this field, sensitivity analysis confirms no significant impact of small samples); (2) Exclusion Criteria: ① Non-randomized controlled trials (e.g., case reports, reviews); ② Failure to clearly report N200/P300 wave amplitudes in ERP; ③ Non-English literature; ④ Methodological flaws (small sample size, unclear randomization methods, lack of control groups); (3) Grouping Method: Groups are formed based on "ERP component types (P300/N200)" and "task mechanisms (e.g., P300 subgroups: anticipatory-response regulation, conscious spatial cognition, unconscious inhibition; N200 subgroups: conscious conflict monitoring, unconscious automatic processing, conscious response inhibition)." |
| Information sources | 6 | Specify all databases, registers, websites, organisations, reference lists and other sources searched or consulted to identify studies. Specify the date when each source was last searched or consulted. | 1.1 Literature Search Section (1) Retrieval Tool: NoteExpress software (2) Databases: PubMed, Web of Science (3) Other sources: Not mentioned; registration databases; institutional websites; or literature tracing (only database searches)(4) Retrieval period: January 2016 – July 2025 (All databases were last searched in July 2025) |
| Search strategy | 7 | Present the full search strategies for all databases, registers and websites, including any filters and limits used. | 1.1 Literature Search Section (1) Keyword combinations: ("Badminton" OR "Tennis" OR "Table tennis") AND ("Event-related potential" OR "P300" OR "N200") (2) Screening criteria: No additional filters (e.g., species or document types). Initial screening through title/abstracts excluded reviews, theoretical studies, and survey research. Full-text screening excluded studies not meeting inclusion criteria. |
| Selection process | 8 | Specify the methods used to decide whether a study met the inclusion criteria of the review, including how many reviewers screened each record and each report retrieved, whether they worked independently, and if applicable, details of automation tools used in the process. | 1.3 Literature Search and Screening Process Section and Figure 2 (1) Screening Process: ① Initial screening: Based on title and abstract screening, exclude irrelevant topics, animal studies, and literature not meeting inclusion criteria (e.g., reviews); ② Full-text screening: Obtain full texts of the 15 qualified articles from initial screening, assess eligibility; ③ Finally include 10 studies (2) Reviewer Arrangement: Two researchers (Liang Dai, Wenqing Hu) independently conduct title/abstract screening and full-text screening. Disagreements are resolved through discussion with a third researcher (Yan Gong) (3) Automated Tools: No automated tools were used (4) Results Presentation: The literature screening process is detailed in Figure 2. |
| Data collection process | 9 | Specify the methods used to collect data from reports, including how many reviewers collected data from each report, whether they worked independently, any processes for obtaining or confirming data from study investigators, and if applicable, details of automation tools used in the process. | 1.4 Literature Coding and 1.5.1 Data Analysis Section (1) Data Collection: Basic characteristics of included studies (author, year), sample composition (number of experimental/control group participants), participant demographics (age, gender), core task types, N200/P300 waveform results (see Table 1) (2) Reviewer Assignment: Two researchers independently extracted and coded data, with discrepancies resolved through discussion (3) Data Validation: No contact was made with original study authors for data verification (all data were extracted from included reports) (4) Automated Tools: Not utilized |
| Data items | 10a | List and define all outcomes for which data were sought. Specify whether all results that were compatible with each outcome domain in each study were sought (e.g. for all measures, time points, analyses), and if not, the methods used to decide which results to collect. | 1.2.1 Inclusion Criteria (3) Table 1 and Sections 2.1/2.2 (1) Outcomes: The primary outcome measures the amplitude of the N200 component (μV) and P300 component (μV) in ERP recordings. Secondary outcomes include latency periods (not explicitly mentioned in the original text but only reported for components with amplitude measurements in Table 1 and results). (2) Data Collection Scope: All studies meeting the criteria "netted exercise + ERP detection of N200/P300" were included, with no exclusions based on time points or analytical methods (e.g., studies involving different training durations [e.g., 12 weeks] or task paradigms). |
|  | 10b | List and define all other variables for which data were sought (e.g. participant and intervention characteristics, funding sources). Describe any assumptions made about any missing or unclear information. | Table 1 and Section 1.4 "Literature Coding" (1) Other Variables: ① Participant characteristics (age, gender, group type: athlete vs non-athlete / training group vs control group); ② Intervention characteristics (sport type: badminton / table tennis / tennis; training duration, e.g., Liu T 2017's 12 weeks); ③ Core task type (e.g., SST task, Posner cue paradigm); ④ Research funding source (all included studies did not mention, the original "Funding" section states this study had no external funding). (2) Missing Information Handling: Only Peng Z 2024 did not report participant age. It is assumed this omission does not affect overall effect analysis (sensitivity analysis did not indicate significant interference from this study on results). |
| Study risk of bias assessment | 11 | Specify the methods used to assess risk of bias in the included studies, including details of the tool(s) used, how many reviewers assessed each study and whether they worked independently, and if applicable, details of automation tools used in the process. | (1) Bias risk assessment tool: The Newcastle-Ottawa Scale (NOS) was employed to assess the methodological quality of the included cross-sectional studies, covering three domains: selection (4 points), comparability (2 points), and outcome ascertainment (3 points), with a total score of 9 (7-9 high, 4-6 moderate, 0-3 low quality). (2) Reviewer assignment: Two researchers (Liang Dai, Wenqing Hu) independently assessed the bias risk of each included study; discrepancies were resolved through discussion with a third researcher (Yan Gong). (3) Automated tools: No automated tools were used. (4) Results presentation: Detailed item-by-item NOS scores are provided in the supplementary material. |
| Effect measures | 12 | Specify for each outcome the effect measure(s) (e.g. risk ratio, mean difference) used in the synthesis or presentation of results. | 1.5.5 Effect Size Pooling Section (1) N200 amplitude: Standardized mean difference (SMD, Hedges' g) with 95% confidence intervals was used as the effect size to account for variations in ERP measurement protocols across laboratories. (2) P300 amplitude: Same as N200, using SMD (Hedges' g). |
| Synthesis methods | 13a | Describe the processes used to decide which studies were eligible for each synthesis (e.g. tabulating the study intervention characteristics and comparing against the planned groups for each synthesis (item #5)). | 1.5.1 Data Analysis and 2.1.5/2.2.5 Subgroup Analysis Results (1) P300 Synthesis Eligibility: ① Overall synthesis: included all 8 studies reporting P300 amplitude (9 studies in Table 1 excluding Shi J 2024, with You Y 2018 et al. among the 8 studies reporting P300 amplitude); ② subgroup synthesis: grouped by "task mechanism + consciousness level", Action Expectation-Response Regulation subgroup included Chen J 2019, Liu T 2017, Wang C 2022 (3 studies), Conscious Spatial Cognition subgroup included Chang CL 2024, Peng Z 2022, Peng Z 2024, Wang CH 2017 (4 studies), Unconscious Inhibition subgroup included Chen KF 2024, You Y 2018 (2 studies). (2) N200 Synthesis Eligibility: ① Overall synthesis: included all 5 studies reporting N200 amplitude (Liu T 2017, Chen J 2019, Wang C 2022, Shi J 2024, You Y 2018); ② Sub-assembly: the sub-group of conscious conflict monitoring included Liu T 2017, Wang C 2022 (2), the sub-group of unconscious automatic processing included Shi J 2024, You Y 2018 (2), and the sub-group of conscious response inhibition included Chen J 2019 (1) |
|  | 13b | Describe any methods required to prepare the data for presentation or synthesis, such as handling of missing summary statistics, or data conversions. | 1.5.5 Effect Size Pooling and Table 1 Section (1) Missing Value Handling: All included studies provided complete reports of mean (Mean), standard deviation (SD), and sample size for N200/P300 wave amplitudes. No missing data were aggregated, and no interpolation was required. (2) Data Conversion: No unit or format discrepancies were observed (all in μV format), thus no conversion was necessary. |
|  | 13c | Describe any methods used to tabulate or visually display results of individual studies and syntheses. | Results: Partial Figures and Table 1 (1) Individual Study Results: Summarized core characteristics and N200/P300 amplitude results of 10 studies in Table 1; Forest plots (Figure 4, Figure 8) displaying effect sizes and 95% confidence intervals (CI) for individual studies. (2) Synthesis Results: ① Forest plots (Figure 4: Overall P300 synthesis; Figure 6: Subgroup P300 synthesis; Figure 8: Overall N200 synthesis; Figure 10: Subgroup N200 synthesis) present aggregated effect sizes; ② Funnel plots (Figure 3: P300 publication bias; Figure 7: N200 publication bias; Figure 5: Subgroup P300 publication bias; Figure 9: Subgroup N200 publication bias) visualize distribution patterns. |
|  | 13d | Describe any methods used to synthesize results and provide a rationale for the choice(s). If meta-analysis was performed, describe the model(s), method(s) to identify the presence and extent of statistical heterogeneity, and software package(s) used. | 1.5.3 Heterogeneity Testing, 1.5.5 Effect Size Pooling and 2.1.2/2.2.2 Section (1) Synthesis Method: Meta-analysis was employed as all included studies were cross-sectional and quasi-experimental studies with consistent outcome measures. 2) Model Selection: ① Heterogeneity testing: Assessed using Q-test (χ² test), I² statistic, and Tau² statistic (Q-test P<0.05, I²>50%, Tau²>0.2 indicate significant heterogeneity). ② Model selection: Random effects model was used when significant heterogeneity was present, and fixed effects model when no significant heterogeneity was observed. 3) Heterogeneity evaluation methods: Q-test (e.g., P300 overall Q=182.87, df=8, P<0.00001), I² statistic (e.g., N200 overall I²=97%), Tau² statistic (e.g., P300 overall Tau²=5.04).4) Software: RevMan 5.4 was used for analysis. |
|  | 13e | Describe any methods used to explore possible causes of heterogeneity among study results (e.g. subgroup analysis, meta-regression). | 1.5.1 Data Analysis and 2.1.5/2.2.5 Subgroup Analysis Results (1) Heterogeneity Exploration Methods: Task-specific mechanism subgroup analysis was employed (as heterogeneity primarily stems from "task-specific mechanisms", as mentioned in sections 2.1.2 and 2.2.2), while meta-regression was not used due to insufficient sample size (10 studies) for regression analysis. (2) P300 Heterogeneity Exploration: Three subgroups were formed based on "task type + consciousness level" (action expectation-response regulation, conscious spatial cognition, unconscious inhibition). The results showed significantly reduced heterogeneity within subgroups (e.g., I subgroup with conscious spatial cognition ²=0%). (3) N200 Heterogeneity Exploration: Three subgroups were formed based on "conflict processing type + consciousness level" (conscious conflict monitoring, unconscious automatic processing, conscious response inhibition). The results indicated 0% heterogeneity within these subgroups. |
|  | 13f | Describe any sensitivity analyses conducted to assess robustness of the synthesized results. | 1.5.4 Sensitivity Analysis and 2.1.4/2.2.4 Section (1) Sensitivity Analysis Method: The "single study exclusion" method was employed, where each study excluded one by one. After excluding each study, the standardized mean difference (SMD), heterogeneity index (I²), and overall effect significance (p-value) were recalculated to assess individual studies 'impact on the overall results. (2) Software: RevMan 5.4 (3) Analysis Content: ① P300: After excluding individual studies, the MD fluctuated between 0.98-2.62, with I² ranging from 40% to 96%, p<0.05 (overall positive effect remained stable); ② N200: Excluding Chen J's 2019 study (conscious response inhibition subgroup) reduced I² from 97% to 0%, while the MD became a significant negative effect, indicating this study was the core source of N200 heterogeneity. |
| Reporting bias assessment | 14 | Describe any methods used to assess risk of bias due to missing results in a synthesis (arising from reporting biases). | 1.5.2 Publication Bias 2.1.1/2.2.1 Part (1) Reporting Bias Evaluation Method: ① Egger regression test (quantitative analysis); ② funnel plot (qualitative visualization). (2) Specific Procedures: ① Conduct Egger tests separately for overall synthesis and subgroup synthesis of P300 and N200 data to calculate intercept t-values and P-values; ② Plot funnel plots to assess symmetry in effect size distribution (symmetry indicates unbiased results, asymmetry suggests biased outcomes). |
| Certainty assessment | 15 | Describe any methods used to assess certainty (or confidence) in the body of evidence for an outcome. | (1) The GRADE (Grading of Recommendations Assessment, Development and Evaluation) method was not used to evaluate evidence certainty. Due to the small number of included studies (P300=8 items, N200=5 items), some studies had methodological limitations (e.g., small sample size, no report of blinding). The evaluation of evidence certainty needs to be supplemented in future studies with expanded sample sizes. |
| **RESULTS** | | |  |
| Study selection | 16a | Describe the results of the search and selection process, from the number of records identified in the search to the number of studies included in the review, ideally using a flow diagram. | 1.3 Literature Search and Screening Process (Figure 2(1)) Results: A total of 15,763 records were retrieved from PubMed and Web of Science. 2) Screening Process: ① Deduplication: Removed 15 duplicate records; ② Initial screening (title/abstract): Eliminated 15,733 irrelevant, animal studies, or non-compliant records, leaving 15 records; ③ Full-text screening: Removed 5 records (3 duplicates, 1 with unreliable data, 1 review), ultimately including 10 studies. 3) Flowchart: See Figure 2 (Literature Search and Screening Process). |
|  | 16b | Cite studies that might appear to meet the inclusion criteria, but which were excluded, and explain why they were excluded. | (1) Excluded Papers: A total of 5 papers were excluded during the full-text screening stage (consistent with Figure 2 in P300 和 N200 Meta analyse.docx), with reasons categorized as: ① Duplicate data with included studies (n=3); ② Unclear reporting of original N200/P300 amplitude data and statistical parameters (unreliable data, n=1); ③ Review articles without original data (n=1). (2) Excluded Studies: No separate citations were provided for excluded studies, as they did not meet the core inclusion criteria (non-randomized controlled trials or lack of ERP data) and were not relevant to the final analysis. |
| Study characteristics | 17 | Cite each included study and present its characteristics. | 1.4 Literature Coding and Table 1 (1) Included studies: 10 studies [1]-[10] (Specific references: [1] Liu T 2017; [2] Chen J 2019; [3] Wang CH 2017; [4] Chang CL 2024; [5] Wang C 2022; [6] Chen KF 2024; [7] Peng Z 2024; [8] Peng Z 2022; [9] You Y 2018; [10] Shi J 2024) (2) Study characteristics: Presented in Table 1, including sample composition (e.g., Liu T 2017:20 participants in the badminton training group vs. 20 in the control group), participant demographics (e.g., age 23-27 years, male), core task types, and N200/P300 waveform results |
| Risk of bias in studies | 18 | Present assessments of risk of bias for each included study. | Supplementary Materials Table S1 (1) Bias Risk Distribution: ① Random Sequence Generation: Most studies did not explicitly describe (bias risk "unknown"); ② Assignment Concealment: All studies did not report (bias risk "unknown"); ③ Blinding: Due to the intervention being exercise training, blinding subjects was difficult (bias risk "high"), but blinding of outcome assessors was not reported ("unknown"); ④ Outcome Data Completeness: All studies had no missing data ("low"); ⑤ Selective Reporting: All reported N200/P300 amplitudes without selective reporting ("low"). (2) Presentation Format: Each study's bias risk (low/high/unknown) across dimensions is presented in a table (Supplementary Material Table S1). |
| Results of individual studies | 19 | For all outcomes, present, for each study: (a) summary statistics for each group (where appropriate) and (b) an effect estimate and its precision (e.g. confidence/credible interval), ideally using structured tables or plots. | Table 1 and Figures 4, 8(1) N200 amplitude (5 studies): ① Group summary statistics: Table 1 reported mean amplitudes for experimental vs. control groups (e.g., Chen J 2019: Experimental group-0.55μV, control group-1.72μV); ② Effect estimation: Figure 8 (N200 forest plot) reported standardized mean difference (SMD) and 95% confidence interval (CI) for individual studies (e.g., Liu T 2017: MD= -1.30,95% CI [-2.80,0.20]) (2) P300 amplitude (8 studies): ① Group summary statistics: Table 1 reported mean amplitudes for experimental vs. control groups (e.g., You Y 2018: Experimental group 22.98μV, control group 16.57μV); ② Effect estimation: Figure 4 (P300 forest plot) reported mean difference (MD) and 95% CI for individual studies (e.g., You Y 2018: MD = 6.41,95% CI [5.46,7.36]) |
| Results of syntheses | 20a | For each synthesis, briefly summarise the characteristics and risk of bias among contributing studies. | 2.1.3/2.1.5/2.2.3/2.2.5 Subgroups (1) P300 Whole Synthesis (8 studies): ① Characteristics: Exercise types included badminton (4), table tennis (3), and tennis (1); participants aged 18-28, predominantly male; ② Risk of bias: Most studies had unknown random sequence generation/distribution, high risk of blinding, and complete outcome data (low risk). (2) P300 Subgroup Synthesis: ① Action-Expectation-Response Regulation subgroup (3 studies): All badminton/tennis research, same risk as above; ② Conscious spatial cognition subgroup (4 studies): Badminton/table tennis research, same risk as above; ③ Unconscious inhibition subgroup (2 studies): All table tennis research, same risk as above. (3) N200 Whole Synthesis (5 studies): ① Characteristics: Exercise types included badminton (2), table tennis (2), and tennis (1); ② Risk of bias: Consistent with P300 studies (4). (4) N200 Subgroup Synthesis: ① Conscious conflict monitoring subgroup (2 studies): Badminton/tennis research; ② Unconscious automatic processing subgroup (2 studies): Table tennis research; ③ Conscious response inhibition subgroup (1 study): Badminton research, same risk as above. |
|  | 20b | Present results of all statistical syntheses conducted. If meta-analysis was done, present for each the summary estimate and its precision (e.g. confidence/credible interval) and measures of statistical heterogeneity. If comparing groups, describe the direction of the effect. | 2.1.3/2.1.5/2.2.3/2.2.5 Partial and Individual Forest Plots (1) P300 Synthesis: ① Overall synthesis: MD=2.22,95% CI [0.62,3.82], P=0.006 (significant positive effect; exercise training increases P300 amplitude), heterogeneity I²=96%, Tau²=5.04 (Figure 4); ② Subgroup synthesis: Action Expectation-Response Regulation subgroup MD=0.02,95% CI [-0.23,0.28], P=0.85 (no significant effect), I²=0%; Conscious Spatial Cognition subgroup MD=1.74,95% CI [0.86,2.62], P<0.0001 (significant positive effect), I²=0%; Unconscious Inhibition subgroup MD=6.42,95% CI [5.50,7.33], P<0.00001 (extremely significant positive effect), I²=0% (Figure 6). (2) N200 Synthesis: ① Overall synthesis: MD=-0.86,95% CI [-2.28,0.56], P=0.24 (no significant effect), heterogeneity I²=97%, Tau²=2.46 (Figure 8). ② Subgroup Analysis: Conscious Conflict Monitoring (MD= -1.66,95% CI [-1.95, -1.37], P <0.00001; significant negative effect of exercise training reducing N200 amplitude), I² = 0%; Unconscious Automatic Processing (MD= -1.29,95% CI [-1.77, -0.81], P <0.00001; significant negative effect), I² = 0%; Conscious Response Inhibition (MD = 1.17,95% CI [0.81,1.53], P <0.00001; significant positive effect), I² not applicable (Figure 10) |
|  | 20c | Present results of all investigations of possible causes of heterogeneity among study results. | 2.1.2/2.1.5/2.2.2/2.2.5 Part (1) P300 Heterogeneity Source: ① Overall heterogeneity I²=96%, with the core source being "task mechanism differences" (subgroup analysis showed I²=0% within subgroups with identical task types). Specific differences: Conscious spatial cognition tasks (P300 increase) vs action expectation-response regulation tasks (no effect) vs unconscious inhibition tasks (significant P300 increase), related to "conscious level (conscious vs unconscious)" and "neural circuits (parietal-frontal network vs basal ganglia-frontal network)". (2) N200 Heterogeneity Source: ① Overall heterogeneity I²=97%, with the core source being "conflict processing type differences" (subgroup analysis showed I²=0% within subgroups). Specific differences: Conscious response inhibition tasks (N200 increase) vs conscious conflict monitoring/unconscious automatic processing tasks (N200 decrease), related to "behavioral output types (active inhibition vs passive monitoring)" and "neural circuits (frontal-basal ganglia network vs prefrontal-cingulate network)". |
|  | 20d | Present results of all sensitivity analyses conducted to assess the robustness of the synthesized results. | 2.1.4/2.2.4 Subsection (1) P300 Sensitivity Analysis: ① Excluding individual studies, the MD fluctuated between 0.98-2.62 with a 95% CI that never crossed zero, and P<0.05 (overall positive effect remained stable). ② Heterogeneity I² fluctuated between 40%-96% (still indicating moderate-to-high heterogeneity, but not affecting the direction of effects). (2) N200 Sensitivity Analysis: ① Excluding Chen et al. (2019) (conscious response inhibition subgroup), the MD= -1.35 with a 95% CI [-1.78, -0.92], P<0.00001 (significant negative effect), and I² decreased to 0%. ② After excluding other studies, the MD remained negative (-0.91 to-1.28), with I² maintained at 97% (indicating Chen et al. (2019) was the core source of heterogeneity). |
| Reporting biases | 21 | Present assessments of risk of bias due to missing results (arising from reporting biases) for each synthesis assessed. | 2.1.1/2.2.1 Subgroups and Funnel Plots (1) P300 Synthesis: ① Overall synthesis: Egger test t=-3.479, P=0.010 (significant publication bias). The funnel plot (Figure 3) is asymmetrical (small sample studies deviate from the overall trend), with model explanatory power R²=23.3% (23.3% of effect variation stems from publication bias). ② Subgroup synthesis: The unconscious inhibition subgroup shows symmetrical funnel plots, while other subgroups lack sufficient study numbers (3-4 trials), resulting in inadequate testing power. (2) N200 Synthesis: ① Overall synthesis: Egger test t=0.539, P=0.628 (no significant publication bias). The funnel plot (Figure 7) exhibits mild asymmetry (due to inclusion of only 5 studies, limiting testing power). Subgroup synthesis: With limited study numbers (1-2 trials), no publication bias test was performed. |
| Certainty of evidence | 22 | Present assessments of certainty (or confidence) in the body of evidence for each outcome assessed. | (1) P300 amplitude: ① Evidence certainty "moderate": Overall effect is significant, but with publication bias (potentially overestimating the effect), low subgroup heterogeneity (indicating clear mechanisms); ② Limitations: Small sample size of included studies (10-25 participants per group), unknown partial bias risk. (2) N200 amplitude: ① Evidence certainty "low": No significant overall effect, limited number of studies (only 5 included). Although no publication bias exists, significant subgroup differences require further validation. ② Limitations: High proportion of unknown bias risks and lack of long-term training effect data. |
| **DISCUSSION** | | |  |
| Discussion | 23a | Provide a general interpretation of the results in the context of other evidence. | 3. Conclusion Section (1) Result Interpretation: The regulation of N200/P300 by netted movement demonstrates "task mechanism specificity": ① The positive effect of P300 primarily stems from conscious spatial cognition (enhanced activation of the parietal network) and the unconscious inhibition task of table tennis (optimization of the basal ganglia-forebrain network), consistent with Polich J's (2007) theory that "P300 reflects attention allocation and decision-making"; ② The differentiation of N200 effects correlates with "conflict processing types" (active inhibition requires more neural resources → increased wave amplitude; passive monitoring/unconscious processing improves neural efficiency → reduced wave amplitude), supporting Yeung N's (2004) "conflict monitoring theory". (2) Connection with Existing Evidence: This study supplements evidence in the "motor-cognitive neuroplasticity" field, explaining the inconsistency in previous research conclusions (failure to consider differences in task mechanisms). |
|  | 23b | Discuss any limitations of the evidence included in the review. | 3. Conclusion (1) Limitations of the Study: ① Small sample size (most studies had 10-20 participants per group), which may affect the stability of effect sizes; ② Uneven types of sports (most studies focused on badminton/tennis, with only one study on tennis), making it difficult to generalize to all racquet sports; ③ Lack of long-term training effects (only Liu et al. (2017) reported 12 weeks of training, while others were cross-sectional studies); ④ Publication bias in P300 measurements (positive results are more likely to be published), which may overestimate true effects. |
|  | 23c | Discuss any limitations of the review processes used. | 3. Conclusion (1) Limitations of the Review Process: ② The search database only included PubMed and Web of Science, excluding platforms like Cochrane Library and Embase, which could result in missed studies; ③ Inadequate reporting of bias risk assessments (e.g., blinding procedures), potentially compromising the credibility of findings; ④ Failure to conduct meta-regression analysis, making it impossible to quantify other sources of heterogeneity (such as age, training intensity). |
|  | 23d | Discuss implications of the results for practice, policy, and future research. | 3. Conclusion (1) Practical Significance: Provides guidance for optimizing specialized training (e.g., focusing on spatial cognition tasks in badminton training, or enhancing unconscious inhibition tasks in table tennis training); Offers neurophysiological basis for "exercise intervention cognitive impairment" (2) Future Research Directions: ① Expand sample size and increase tennis research to balance exercise types; ② Refine subgroups (e.g., by training duration and intensity) for meta-regression analysis; ③ Correct publication bias using trimming and filling methods; ④ Conduct long-term follow-up studies to explore the persistence of training effects; ⑤ Register review protocols and improve risk assessment of biases. |
| **OTHER INFORMATION** | | |  |
| Registration and protocol | 24a | Provide registration information for the review, including register name and registration number, or state that the review was not registered. | (1) Registration information: This meta-analysis was registered on PROSPERO (International Prospective Register of Systematic Reviews), registration number CRD420261421340. (2) Protocol: No study protocol was prepared prior to the review, and no protocol amendments were made (as no initial protocol existed). (3) Transparency note: All inclusion/exclusion criteria and analysis methods were pre-defined based on PRISMA 2020 guidelines and reported in detail in the "Methods" section of the main manuscript. |
|  | 24b | Indicate where the review protocol can be accessed, or state that a protocol was not prepared. | No study protocol was prepared for this meta-analysis, and therefore no protocol is available |
|  | 24c | Describe and explain any amendments to information provided at registration or in the protocol. | No amendments were made to the registered review information. |
| Support | 25 | Describe sources of financial or non-financial support for the review, and the role of the funders or sponsors in the review. | Manuscript "Funding" (1) Funding support: This research did not receive any external funding (2) Non-funding support: The author's unit (Jiangxi Science and Technology Normal University, Nanjing School of Nanchang) provided research space and literature retrieval resources. The sponsor did not participate in the research design, data collection, analysis, result interpretation and paper writing |
| Competing interests | 26 | Declare any competing interests of review authors. | Manuscript “Competing Interests”  The authors declare that they have no competing interests |
| Availability of data, code and other materials | 27 | Report which of the following are publicly available and where they can be found: template data collection forms; data extracted from included studies; data used for all analyses; analytic code; any other materials used in the review. | Manuscript Corresponding Author Information and Supplementary Materials (1) Data Extraction Form: Table 1 summarizes the core data included in the study. The complete data extraction form (including incubation period and sample size details) can be obtained by contacting the author (Yan Gong (corresponding author), Email: 26230232@qq.com). (2) Analytical Data: Original data used for meta-analysis (e.g., Mean, SD, sample size of each study) and summary analysis results can be obtained through the author. (3) Analysis Code: The analysis was conducted using RevMan 5.4 without additional coding. (4) Other Materials: PRISMA checklist, PRISMA flowchart (Revised Version Figure 2), and bias risk assessment table (Supplementary Material Table S1) are submitted as supplementary files with the revised manuscript. |
