# Supplementary material for: Meta-analysis of task- and mechanism-dependent effects of net sports on N200 and P300 components
Source: Front Psychol. 2026 Sep 25;17:1903090. doi: 10.3389/fpsyg.2026.1903090 (PMC13649459; doi:10.3389/fpsyg.2026.1903090)
Supplement: Supplementary file 2 [file Table_2.docx]

Supplementary B: Risk of Bias Assessment Table (Cochrane Tool). This table summarizes the risk of bias evaluation results for 10 included randomized controlled trials. Assessment dimensions include random sequence generation, allocation concealment, blinding (participants/researchers/outcome assessors), data completeness, selective reporting, and other biases (study registration, conflict of interest). Risk levels are labeled as 'Low risk', 'High risk', or 'Unknown', with rationales based on the original study’s methodological descriptions.

| Included Studies (Author + Year + Core Project) | Random Sequence Generation (Risk/Basis) | Allocation Concealment (Risk/Basis) | Blinding of Participants and Researchers (Risk/Basis) | Blinding of Outcome Assessors (Risk/Basis) | Data Completeness (Risk/Basis) | Selective Reporting (Risk/Basis) | Other Biases (Study Registration/Conflicts of Interest) |
| --- | --- | --- | --- | --- | --- | --- | --- |
| 1. Chen et al. (2024) [Table Tennis] | High risk (Grouped by sports level; no randomization method mentioned) | Unclear (No relevant description) | High risk (Only the study purpose was concealed; participants were aware of group allocation) | Unclear (No relevant description) | Low risk (All 54 participants included; ERP amplitude exceeding ±50mV excluded) | Low risk (Complete reporting of behavioral indicators + P3 indicators) | Unregistered; Hung is an editor of PeerJ; no other conflicts of interest |
| 2. Peng et al. (2024) [Table Tennis] | High risk (Grouped by athlete/student identity; non-randomized) | Unclear (No relevant description) | High risk (Participants were aware of group allocation by identity; no blinding) | Unclear (No relevant description) | Low risk (8 cases of artifacts excluded; 40 participants finally included) | Low risk (Complete reporting of behavioral indicators + ERP + functional connectivity) | Unregistered; no conflicts of interest |
| 3. Peng et al. (2022) [Table Tennis] | High risk (Grouped by athlete/student identity; non-randomized) | Unclear (No relevant description) | High risk (Participants were aware of group allocation by identity; no blinding) | Unclear (No relevant description) | Low risk (2 cases of artifacts excluded; 48 participants finally included) | Low risk (Complete reporting of behavioral indicators + P3 hemispheric differences) | Unregistered; no conflicts of interest |
| 4. Liu et al. (2017) [Badminton] | Unclear (Mentioned "random grouping" but no method specified) | Unclear (No relevant description) | High risk (Only the study purpose was concealed; participants were aware of training/control groups) | Unclear (No relevant description) | Low risk (4 cases of artifacts excluded in each group; 32 participants finally included) | Low risk (Complete reporting of behavioral IES + N2/P3 indicators) | Unregistered; no conflicts of interest |
| 5. Chang et al. (2024) [Badminton] | High risk (Grouped into elite/expert/intermediate based on competition performance; non-randomized) | Unclear (No relevant description) | High risk (Participants were aware of group allocation by competitive level; no blinding) | Unclear (No relevant description) | Low risk (All 54 participants completed the study; incorrect trials excluded) | Low risk (Complete reporting of RT/AR + N2/P3 indicators) | Unregistered; no conflicts of interest |
| 6. Wang & Tu (2017) [Badminton vs. Track and Field] | High risk (Grouped by sports event; non-randomized) | Unclear (No relevant description) | High risk (Participants were aware of group allocation by sports event; no blinding) | Unclear (No relevant description) | Low risk (All 32 participants completed the study; ERP artifacts exceeding ±100μV excluded) | Low risk (Complete reporting of RT + CNV/P3 indicators) | Unregistered; no conflicts of interest |
| 7. Chen et al. (2019) [Badminton] | High risk (Grouped by athlete/non-athlete identity; non-randomized) | Unclear (No relevant description) | High risk (Participants were aware of group allocation by identity; no blinding) | Unclear (No relevant description) | Low risk (2 outliers excluded; 39 participants finally included) | Low risk (Complete reporting of RT/SSRT + N1/N2/P3 indicators) | Unregistered; no conflicts of interest |
| 8. Chen et al. (2024) [Multiple Sports] | High risk (Grouped into elite/amateur/non-athlete based on sports level; non-randomized) | Unclear (No relevant description) | High risk (Only the study purpose was concealed; participants were aware of group allocation) | Unclear (No relevant description) | Low risk (All 54 participants included; ERP amplitude exceeding ±50mV excluded) | Low risk (Complete reporting of behavioral indicators + P3 indicators) | Unregistered; no conflicts of interest |
